# Enriched methylomes of low-input and fragmented DNA using fragment ligation EXclusive methylation sequencing

**DOI:** 10.1093/nar/gkag385

**Published:** 2026-05-14

**Authors:** Jingru Yu, Lauren S Ahmann, Yvette Y Yao, Angus Toland, Alicia Snowden, Chandler Ho, Netanel Loyfer, Tommy Kaplan, Hannes Vogel, Linlin Wang, Brooke E Howitt, Brittany Holmes, Alarice Cheng-Yi Lowe, Wei Gu

**Affiliations:** Department of Pathology, School of Medicine, Stanford University, Stanford, CA 94305, United States; Department of Pathology, School of Medicine, Stanford University, Stanford, CA 94305, United States; Department of Pathology, School of Medicine, Stanford University, Stanford, CA 94305, United States; Department of Pathology, School of Medicine, Stanford University, Stanford, CA 94305, United States; Children’s Hospital Colorado, University of Colorado Anschutz Medical Campus, Aurora, CO 80045, United States; College of Medicine, Howard University, Washington, DC 20059, United States; Clinical Laboratories, Stanford Health Care, Stanford, CA 94305, United States; School of Computer Science and Engineering, The Hebrew University of Jerusalem, Jerusalem 9190501, Israel; School of Computer Science and Engineering, The Hebrew University of Jerusalem, Jerusalem 9190501, Israel; Faculty of Medicine, The Hebrew University of Jerusalem, Jerusalem 9112001, Israel; Department of Pathology, School of Medicine, Stanford University, Stanford, CA 94305, United States; Department of Pediatrics, School of Medicine, Stanford University, Stanford, CA 94305, United States; Department of Laboratory Medicine, School of Medicine, University of California San Francisco, San Francisco, CA 94143, United States; Department of Pathology, School of Medicine, Stanford University, Stanford, CA 94305, United States; Department of Pathology, School of Medicine, Stanford University, Stanford, CA 94305, United States; Department of Pathology, School of Medicine, Stanford University, Stanford, CA 94305, United States; Department of Pathology, School of Medicine, Stanford University, Stanford, CA 94305, United States

## Abstract

Epigenetic profiling is an emerging clinical tool for tumor profiling and liquid biopsies. Here, we developed Fragment Ligation EXclusive methylation sequencing (FLEXseq), a genome-wide methylation profiler that enriches and sequences the DNA fragments flanking CCGG motifs to cover a broad range of regulatory regions. FLEXseq strongly correlates with whole genome bisulfite sequencing (WGBS; Pearson’s *r* = 0.97) yet requires only three- to seven-fold less sequencing depth than whole genome approaches to cover cell type-specific markers and 4- to 20-fold less sequencing to cover key regulatory regions like promoters. DNA dilutions down to 250 pg decreased CpG coverage, but bias in methylation remained low (Pearson’s *r* ≥ 0.90). To demonstrate the broad applicability of FLEXseq, including highly fragmented DNA found in clinical specimens, we verified its usage across cells, body fluids, and formalin-fixed paraffin-embedded (FFPE) tissues. Across 106 cerebrospinal fluids, a specimen type challenged by low-input and fragmented DNA, FLEXseq enabled cell type deconvolution to distinguish between different tumor types and negative controls with an accuracy of 97%. FLEXseq offers a cost-efficient, single-nucleotide resolution approach to profile the methylome even with fragmented, low-input DNA.

## Graphical Abstract

**Graphical Abstract:**
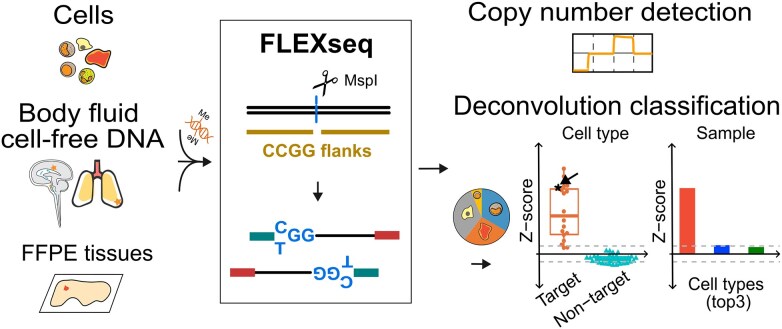

## Introduction

Highly multiplexed DNA methylation testing is rapidly advancing, fueled by the growing interest in early cancer detection [1–4] and the clinical adoption of tumor classification [5–9]. Further applications are emerging in diverse fields, such as aging through epigenetic clocks [10] and liquid biopsies based on cell type deconvolution [11–13]. Methylation markers provide stable epigenetic data on DNA preserved in clinical specimens like cell-free DNA (cfDNA) from body fluids and fragmented DNA from formalin-fixed paraffin-embedded (FFPE) tissues.

Whole-genome bisulfite sequencing (WGBS) is the gold standard for methylation profiling. However, it is not routinely used at high coverage or at scale as it is cost-prohibitive. Targeted methods often require *a priori* knowledge of evolving markers that are expanding and differ across unreferenced tumor and cell types. See the Discussion for details on the strengths and limitations of methylation profiling methods.

**Figure 1.**
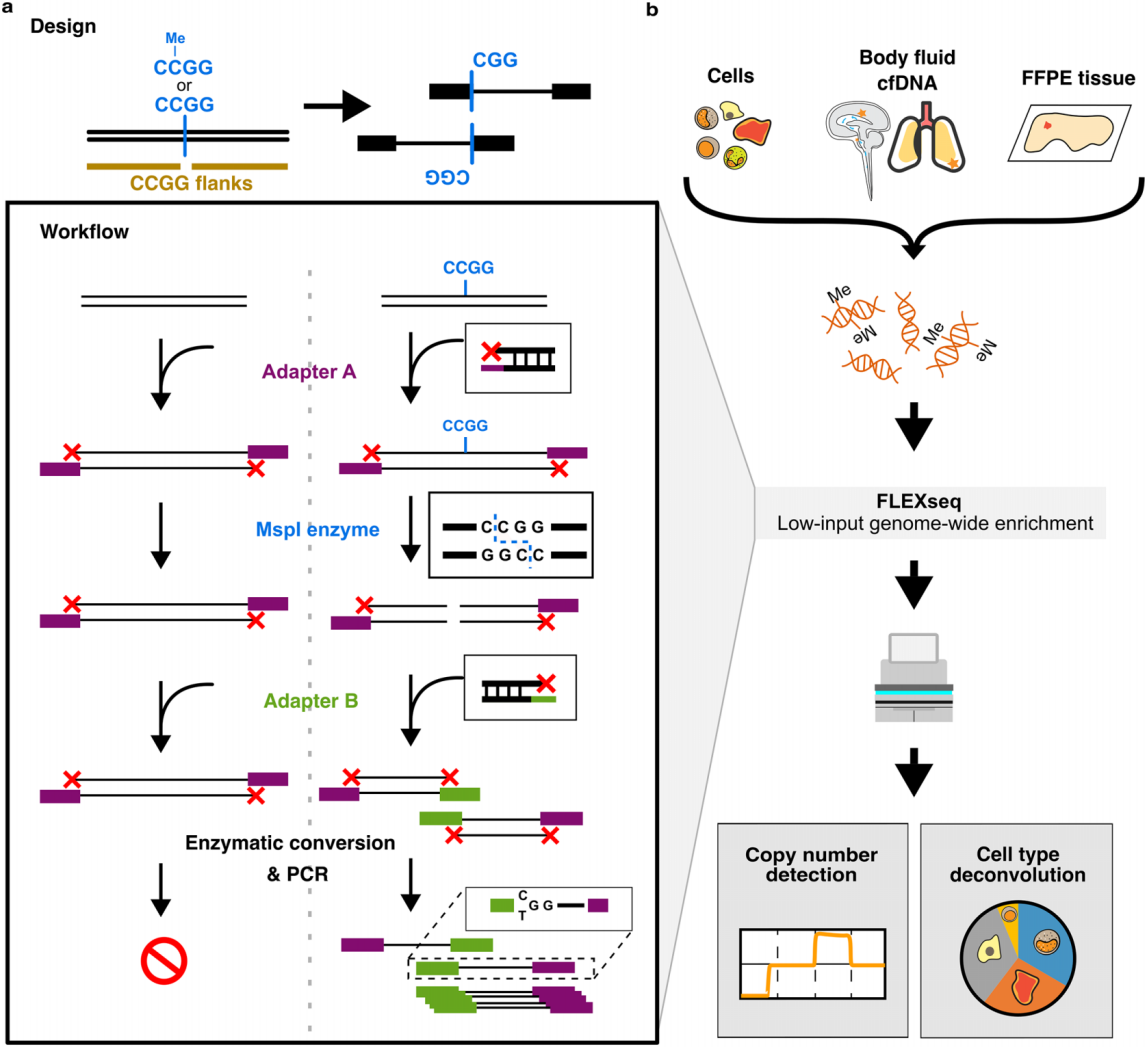
Schematic of FLEXseq design and workflow. (**a**) The design goal and workflow of FLEXseq. The design aims to sequence adjacent regions flanking CCGG motifs while preserving the methylation markers at the motif. Information-poor regions are suppressed, while information-rich CCGG flanking regions are amplified. In the workflow, input DNA fragments are first ligated with a semi-permissive Adapter A, which serves both as an essential blocker for untargeted DNA and a required piece of targeted DNA. The nuclease MspI then cuts at the CCGG motif regardless of methylation status, followed by the ligation of Adapter B. Only molecules with both Adapters A and B are amplified and sequenced. (**b**) The overall workflow and analyses. Specimen inputs are sheared genomic (g)DNA from cells, cfDNA from body fluid and plasma, and fragmented DNA from formalin-fixed paraffin-embedded (FFPE) tissues. Analyses include copy number detection for malignant aneuploidy and deconvolution of cell types.

Here we present FLEXseq (Fragment Ligation EXclusive methylation sequencing), a methylation profiling method that targets the adjacent flanks of CCGG motifs in the genome that associate with cell type-specific markers, promoters, and other epigenetic functional elements using the MspI restriction enzyme (Fig. 1a). MspI-based reduced representation approaches (e.g. RRBS) have long leveraged CCGG digestion to enrich CpG-dense regions, and RRBS classically enriches fragments with two MspI-cut ends [14]. FLEXseq and extended-representation bisulfite sequencing (XRBS) [15] extend the genomic coverage, particularly in fragmented DNA, by capturing fragments with one MspI-cut end and one free end. However, XRBS is impractical in enriching fragmented and low-input DNA found in clinical specimens (e.g. cfDNA, fragmented FFPE tissue DNA). To design for fragmented DNA, we introduced a semi-permissive adapter that selectively blocks the free ends of non-target DNA, allowing FLEXseq to deliver a highly on-target and accurate methylome.

To demonstrate the versatility of FLEXseq, we used cell lines, cfDNA across different body fluids, and fragmented FFPE tissue DNA (Fig. 1b), highlighting the ability to profile an enriched methylome and perform a genome-wide copy number analysis in fragmented DNA. To demonstrate the clinical utility of FLEXseq, we performed a case-control study of 106 cerebrospinal fluids (CSF), applying cell type deconvolution for cancer detection and tumor type classification based on the malignant cell-of-origin (COO).

## Materials and methods

### Ethics statement

Body fluid and FFPE tissue samples from Stanford Healthcare were originally processed by the Stanford Pathology and Clinical Laboratories as part of routine clinical testing, and residual material was retrospectively enrolled through a waiver of consent in a protocol approved by the Stanford Institutional Review Board (IRB 58461). The plasma P2 was collected under informed consent in a protocol approved by the Stanford Institutional Review Board (IRB 71230). IRB approvals and sample collection details from the UCSF Medical Center are detailed in past publications [16–18].

### In silico pre-design analysis

*In silico* CCGG flanks, cell type, array, RRBS, and MeDIP-seq markers: For the *in silico* analysis before designing FLEXseq, we intersected CpGs from cell type markers and CCGG flanks with other markers and methods. Cell type markers are the top 100 markers from all 39 cell types; see the section ‘*Genomic segmentation and identification of cell type-specific markers*’ below for details. For CpGs on the 450k and EPIC arrays, we downloaded the annotation files from https://zwdzwd.github.io/InfiniumAnnotation. For CCGG flanks, we produced all CCGG motif coordinates in a BED format and expanded the CCGG motifs to specific lengths with ‘bedtools slop -b 50 (100/150/200/300)’. RRBS regions are defined as two CCGG motifs within a maximum distance of 50–300 bp of each other, with the command ‘bedtools merge -d 50 (100/150/200/300)’. The RRBS regions shorter than 30 bp were dropped. For the MeDIP-seq markers, we used only cell type-specific hypermethylated markers since the assay pulls down on methylated markers. For the CNS and TCGA classifier markers, we used the top 32 000 and 60 000 probes, respectively.

Annotation of promoters, enhancers, and other methylation regions: We divided the CpG regions according to genomic localization: CpG islands, shores, regions up to 2 kb from CpG islands, shelves, regions from 2 to 4 kb from CpG islands, and open sea, the rest of the genome. Promoters were defined as 1 kb upstream and downstream of all transcription start sites (downloaded from UCSC) of protein-coding genes. Enhancers were downloaded from Fantom5. Introns, coding exons, 5′ untranslated regions (UTR), and 3′ UTRs were parsed from RefSeq annotation. Intergenic regions were the regions excluding promoters, enhancers, exons, untranslated regions, and introns within protein-coding genes. Each called CpG site is counted once: the overlap of a genomic region (promoters, enhancers, coding sequences, untranslated regions, and introns) excludes all previously overlapped sites starting from promoters. The denominator is the total CpGs across genomic regions, and the numerator is the CpGs covered by methylation detection methods.

### Sample DNA extraction

#### Cell lines

DNA from MCF-7 (ACC 115, BRCA), CL-40 (ACC 535, COAD), and DBTRG-05MG (ACC 359, GBM) was obtained from DSMZ (Braunschweig, Germany), and DNA from K562 (DD2011) was obtained from Promega (Madison, USA).

#### Primary cells

Purified immune cells (purity above 95% by flow cytometry) were obtained from IQ Biosciences, and dissociated cells were extracted using the Quick-DNA Miniprep kit (D3020, Zymo Research).

#### Fresh body fluid

Up to 1 mL of DNA was extracted by the Maxwell RSC ccfDNA Plasma Kit (AS1480, Promega) with approximately 47 µL output. For inputs greater than 1 mL, we used the RSC ccfDNA LV kit instead (AS1480, Promega). Extracted DNA was quantified with Qubit 4 Fluorometer (Thermo Fisher Scientific) for samples likely to have high DNA concentrations, such as CSF cfDNA samples with a white blood cell count ≥ 1000 cells/µL or those lacking WBC data; other CSF samples, typically with low DNA yields, were not quantified to conserve sample volume. Extracted DNA was stored in DNA Lobind tubes at −20°C.

#### FFPE

FFPE blocks or previously extracted and frozen (-80°C) FFPE tissue DNA were used. DNA extraction from FFPE blocks was performed using the Maxwell RSC FFPE Plus DNA kit (AS1720, Promega) or QIAamp DNA FFPE Tissue Kit (PN 56404, Qiagen). The clinical molecular pathology lab found no difference in output between the two kits. Extracted DNA was quantified on a spectrometer (Nanodrop, Thermo Fisher Scientific) or Qubit. Frozen DNA was stored in DNA Lobind tubes at -20°C.

### Sequencing library preparation

#### Shearing

DNA from cell lines, immune cells, and FFPE DNA were sheared with acoustics using a 96 AFA-TUBE TPX plate (PN 520291, Covaris) set at 400–500 bp. K562 DNA was sonicated into short-sheared fragments of ∼180 bp and long-sheared fragments of ∼300 bp. The benchmark FFPE tissue (TF725) DNA was sheared with a peak of 330 bp.-6pt

#### Whole genome libraries

We followed past protocols as previously described [16, 17].

#### FLEXseq

FLEXseq was performed using components of the NEBNext Enzymatic Methyl-seq Kit (E7120L, NEB), including the entirety of the conversion module (E7125L, NEB). All reagent usage was halved before the conversion module, and the input was also halved to 25 µL of extracted DNA. For all steps, liquid handling was performed manually in strip tubes or through a semi-automated 96-well plate workflow using a Formulatrix Mantis dispenser (small volume chip), Beckman i5 liquid handler with a 96-head, and an Integra MINI96 pipettor (125 µL). We recommend and optimize our approach for plate automation. We used Mag-Bind TotalPure beads (M1378-02, Omega Bio-tek) for all bead purifications. See the Supplementary Protocol for details.

To place on the first adapter, we used the end repair module but replaced the pre-set adapter with Adapter A. Adapter A was formed by synthesizing and annealing [5′ C3 Spacer phosphoramidite]-ACACTCTTTCCCTACACGACGCTCTTCCGATC-S-T and [5′ Phosphate]-GATCGGAAGAGCGTCGTGTAGGGAAA GAGTGT-[3′ Amino Modifier] with methylated C (IDT). We added 50 µL beads for bead purification and eluted in 17 µL of water.

After adapter A purification, we removed residual phosphates using 1 µL of Shrimp Alkaline Phosphatase (M0371L, NEB) in 2 µL of 10x NEB buffer 2. The incubation was 1 h at 37°C, followed by 65°C for 10 min to quench the phosphatase. This was followed by restriction enzyme cutting using 10 units of MspI (R0106T, NEB), followed by a 30-min incubation. Next, an end repair was performed using 1 µL of Klenow master mix composed of 1.3 µL of Klenow (M0212M, NEB), 2 µL of 10x NEB buffer 2, 5 µL of a master mix of NTPs, and 11.7 µL of water at an incubation of 30°C for 20 min, followed by 37°C for 20 min, and 65°C for 20 min. The mix of NTPs was composed of 40 µL of the 10 mM dATP, 4 µL of the 10 mM dCTP, and 4 µL of the 10 mM dGTP solution (N0446S, NEB). Finally, we ligated 1 µL of adapter B using NEBNext Ultra II Ligation Module (E7595L, NEB). Adapter B was formed by synthesizing and annealing GATCGGAAGAGCACACGTCTGAACTCCAGTC-[3′ Amino Modifier] and GACTGGAGTTCAGACGTGTGCTC TTCCGATC-S-T with methylated C (IDT). We added 36 µL beads for bead purification and eluted in 28 µL of water.

The subsequent enzymatic conversion of unmethylated cytosines to thymine was accomplished as specified by the manufacturer (E7125L, NEB) with the following modifications. The volume of APOBEC was modified to 75% of the manufacturer’s recommended volumes (60 µL). We spiked in 10–50 ng of PhiX DNA (N3021S, NEB) as filler DNA during the TET conversion step and allowed the APOBEC incubation to extend from 3 h to overnight. We used fresh reagents to optimize for high conversion rates.

The product was amplified by up to 24 cycles of PCR using sequencing platform (Illumina) adaptor primers with unique dual indexing (UDI; e.g. E6440L, NEB). We used a NEBNext Q5U Master Mix (M0544S, NEB) with a spike-in of 7.5 µL 100X SYBR Gold (S11494, Thermo Fisher Scientific) to monitor the PCR curve on a QuantStudio 3 real-time PCR thermocycler (A28567, Thermo Fisher Scientific).

#### Whole genome bisulfite sequencing (WGBS)

Dual-indexed sequencing libraries were prepared from sheared and original/unsheared K562 DNA with different input amounts. End repair and adaptor ligation were performed using the NEBNext End Repair/dA-Tailing (E7546L, NEB) and Ligation (E7595L, NEB) module. All reagent volumes were halved according to the manufacturer’s instructions. The adaptor-ligated DNA was subjected to bisulfite conversion using the EZ DNA Methylation-Gold Kit (D5005, Zymo Research). Libraries were then amplified with 18 cycles of PCR using UDI extension primers with the Q5U Master Mix (M0544S, NEB) with SYBR Gold.

#### Whole genome enzymatic methyl-seq (EM-seq)

Dual-indexed sequencing libraries of plasma cfDNA and fragmented FFPE tissue DNA were constructed with the NEBNext Enzymatic Methyl-seq Kit (E7120L, NEB) with the conversion module (E7125L, NEB), following the manufacturer’s instructions. Final library amplification was performed using sequencing platform (Illumina) adaptor primers with UDI extension and NEBNext Q5U Master Mix (M0544S, NEB) with SYBR Gold.

#### Extended-representation bisulfite sequencing (XRBS)

Dual-indexed libraries of sheared and unsheared DNA with different inputs were prepared following the protocol from Shareef *et al*. [15] with the following modifications. We used adapters containing a methylated top strand with an eight-base C-depleted barcode “TGGTATAG” since the wells from the same sample were combined during library preparation. When the input was low, we diluted the adapters (1:5, 2 nM) before using. Bisulfite conversion was performed on streptavidin beads-bound DNA using the EZ DNA Methylation-Gold Kit (D5005, Zymo Research) according to the manufacturer’s protocol. A final library amplification was carried out with 20 cycles of PCR using UDI extension primers.

#### Reduced representative bisulfite sequencing (RRBS)

Sheared or unsheared genomic DNA was digested with MspI (R0106M, NEB), end-repaired, 3′-dA-tailed, and ligated to adapters using the NEBNext Ultra II modules (E7546L and E7595L, NEB). The Adapter-ligated DNA was subjected to bisulfite conversion using the EZ DNA Methylation-Gold Kit (D5005, Zymo Research). After bisulfite treatment and bead clean-up, the DNA was amplified with 20 cycles of PCR using UDI extension primers. We also modified this method for fragmented DNA with enzymatic conversion.

#### Whole genome sequencing (WGS)

WGS was used as a control for aneuploidy detection. We adapted the NEBNext Ultra II DNA Library Prep Kit (E7645L, NEB), following the manufacturer’s protocol. We used the methylated adapters from the NEBNext Enzymatic Methyl-seq Kit (E7120L, NEB) and skipped the USER enzyme step in the Ultra II protocol. Adapter-ligated DNA was amplified for up to 20 cycles using the Q5 master mix, with UDI extension primers.

#### Sequencing

For FLEXseq and XRBS, libraries were sequenced on an Illumina NovaSeq 6000 using 100-cycle S2 kits configured as paired-end 2 × 50 bp. To address the decrease in nucleotide diversity due to the C to T conversions, we spiked in 10–15% PhiX sequencing library DNA (Illumina) or whole genome libraries. Samples were only included in library pooling if their qPCR curve was ≤14 Ct (estimated to the nearest 0.5). For WGBS, EM-seq, RRBS (and cfDNA-adapted), and benchmarked FLEXseq (P2 and TF725), uniquely dual-indexed libraries with spike-ins of whole genome libraries were pooled and clustered on an Illumina NovaSeqX flow cell with 2 × 150 bp paired-end sequencing.

### Computational preprocessing of sequencing data

Paired-end reads of FLEXseq were quality and length trimmed with cutadapt v.4.4 with the following parameters: -a NNAGATCGGAAGAGC -A NNAGATCGGAAGAGC –minimum-length 25. Benchmarked FLEXseq reads were additionally trimmed to 50 bp to match the read length used across multiple methods. High-quality sequencing reads were then aligned to the hg38 reference, lambda, and pUC19 genomes using Bismark v.0.23.0. Sorted alignments were further processed to only maintain uniquely mapped read pairs with alignment score ≥-5 (- -score_min L,0,-0.2), ignoring the quality values of individual bases during the alignment process, and discarding reads with multiple alignments. For the FASTQ files of WGBS and EM-seq, after trimming adapters and an extra 100 bp using the cutadapt command (‘cutadapt -a AGATCGGAAGAGC -A AGATCGGAAGAGC’), paired-end reads were mapped to the human (hg38), lambda, and pUC19 genomes using Bismark. For XRBS data, we used the cutadapt command with parameters “-a GCTCTTCCGATCT - -discard -q 20,20.” Then all read pairs were trimmed and filtered using TrimGalore v.0.6.7 with parameters - -paired - -illumina - -length 25 - -clip_R1 8 - -three_prime_clip_R2 8” to trim adapters, 8-bp barcode, and drop fragments shorter than 25 bp. Moreover, for RRBS FASTQ files, we used TrimGalore to trim adapters, bases with low quality, 2 bp of filled-in artifacts, and an extra 98 bp, with parameters “- -paired - -illumina - -quality 20 - -rrbs - -three_prime_clip_R1 98 - -three_prime_clip_R2 98 - -length 25.” Reads shorter than 25 bp were also discarded.

Incomplete enzymatic conversion affected large portions of the same read, allowing us to filter out reads with unmethylated cytosine in the non-CpG context with the filter_non_conversion function from Bismark. Using this filter, we found that FFPE samples have a higher filter rate (5.1%) than non-FFPE samples (0.7% for sheared DNA and 0.5% for cfDNA, see Supplementary Table S1a). The detection of non-converted DNA molecules highlights an advantage of base-resolution sequencing to correct for incomplete conversions.

After filtering non-CG converted and duplicated reads (except RRBS data), we used the bismark_methylation_extractor function to extract the methylation calls with the option “- -bedGraph.”

The methylation calls of a CpG dinucleotide entity (CpG duplex) from both top and bottom strands, staggered by 1 bp, were merged using the coverage2cytosine function with the parameter - -discordance 50, to increase coverage per paired CpG location and reduce the memory burden in downstream analyses. Then, all single-nucleotide polymorphism (SNP) positions (dbSNP153Common) were removed for sample deidentification using bedtools.

We also used the bam2pat function from wgbs_tools [19] to convert bam files into PAT and BETA files for deconvolution, keeping reads covering at least three CpG sites (- -min_cpg 3). The PAT files preserved fragment-level data and were deidentified by removing SNPs using the mask_pat function. All deidentified PAT files from each sample are available and used for downstream analyses (see Data Availability).

### Analytical benchmarks

Benchmarks were primarily performed on sheared and original/unsheared K562 DNA, plasma cfDNA, and fragmented FFPE tissue DNA. The correlation of methylation profiles between different approaches was tested using overlapping CpG sites with ≥ 15x coverage from K562. Pearson’s correlation coefficient was calculated using the cor.test R function with single-CpG resolution.

For the reproducibility analysis, we sequenced three K562 replicates at 20 ng DNA input across different library preparation and sequencing runs (see details in Supplementary Table S1).

For the enrichment analysis, we randomly subsampled the aggregated reads from K562 DNA WGBS (828M paired-end reads with 2 × 50 bp with trimming) and FLEXseq (658M paired-end reads with 2 × 50 bp), from plasma cfDNA EM-seq (329M paired-end reads) and FLEXseq (56M paired-end reads), and from fragmented FFPE DNA EM-seq (654M paired-end reads) and FLEXseq (96M paired-end reads), down to 10–800M paired-end reads. We used ‘samtools depth -J’ to calculate depths in target regions. When calculating the CpG site-based enrichment, we excluded CpGs with coverage < 10x and then compared the target enrichment between them based on 5 Gbp data. CpG sites intersected with the CCGG flanks, cell type markers (the top 250 UXM markers from WGBS data from a human DNA methylation atlas [12], Supplementary Table S2), and promoters were counted for comparisons. ChrX, chrY, and chrM were excluded.

To evaluate the performance of different DNA amounts, we titrated DNA inputs from 250 ng to 100, 10, 1, and 0.25 ng of one pleural fluid sample (BF3713) and tested their correlations based on overlapping CpGs sites with coverage ≥15x.

### Copy number analysis

We used CNVkit (v0.9.10) [20] to analyze and visualize genome-wide copy numbers of FLEXseq and WGS data. Our inputs into CNVkit were Bismark/Bowtie 2 aligned BAM files that were deduplicated by Bismark based on end positions and fragment lengths (see Supplementary Methods in detail).

### In silico titrations

We mixed methylation profiles from the human DNA methylation atlas [12] samples with different cell type compositions (Supplementary Table S3), including B cell, T cell, monocyte, granulocyte, and/or hepatocyte, colon epithelium, lung alveolar epithelium, neuron, oligodendrocyte, and endothelium. We simulated seven mixtures at proportions of 0%, 0.3%, 1%, 3%, 10%, 40%, and 100% of target cell types, conducting three mixtures with two replicates for each level. Merging, splitting, and mixing of reads were performed using the wgbs_tools functions ‘merge’ and ‘mix_pat’. We then dropped the CpGs that are covered by WGBS but not CCGG motifs flanked by 50 bp to simulate the FLEXseq data. Matching references from the atlas were used, from which the individual mixed-in samples were excluded. The reference and titrated samples are listed in Supplementary Table S4. The identified markers are listed in Supplementary Table S5.

### In vitro DNA titrations

We extracted, sheared, and quantified DNA from four human immune cell types (B cell, T cell, monocyte, and neutrophil) as described above. DNA was diluted to 5 ng/μL each and then mixed at different proportions to simulate benign samples with a high immune background. Specifically, two background mixtures were made: (i) 25% of each cell type and (ii) 15% each of B cells and monocytes and 35% each of T cells and neutrophils. They were then titrated separately with DNA from three tumor cell lines: BRCA, COAD, and GBM (see details in the ‘*Sample DNA extraction’* section). Tumor cell line DNA was titrated from 100% down to final purities of 60%, 30%, 15%, 7.5%, and 0% (Supplementary Table S6).

### Genome segmentation and identification of genomic features

#### Identification of haplotypes

We used the wgbs_tools function “segment” with 205 samples as references (grouped into 39 groups according to their methylation patterns) to segment the genome into 2,804,836 haplotypes blocks that cover at least 3 CpGs (with parameters “- -min_cpg 3 - -max_bp 5000”), excluding chrX, chrY, and chrM. Each haplotype should have homogeneous methylation levels across multiple consecutive CpGs and cell types. The average methylation per haplotype in a sample was calculated by summarizing methylated and unmethylated calls across the entire region and then calculating a single beta value (methylated counts divided by total counts). We further kept the haplotypes with a length of 10–2000 bp, and CpGs in these haplotypes were intersected with the methylation array probes for computational inference.

#### Identification of cell type-specific markers

We performed a one-vs-all comparison to identify differentially methylated segments with a length of 10–2000 bp for each cell type, using the wgbs_tools function “find_markers” (parameters “- -min_cpg 4 - -min_cov 10 - -delta_means .3 - -pval .05”). From the initial list of segments representing cell type-specific markers, we then selected markers with the following criteria: (i) the average methylation ≥ 0.66 of one cell type and < 0.33 in all others, or vice versa, and (ii) coverage of 30x or more (across all reads that cover CpGs of the marker). We selected the top 30 and 100 markers with the highest delta of beta values for each cell type (for cell types with more than 1000 markers, we further used parameters “delta_means > 0.4” and “delta_quants > 0.1”). Hypomethylated markers were defined based on the difference between the 75th percentile of the segment average methylation within the target samples and the 2.5th percentile of the background samples. Hypermethylated markers were defined based on the difference between the 97.5th percentile of the background and the 25th percentile within the target samples. All filtered, and the top markers were used for *in silico* analyses.

For cell type-specific markers across immune cells (B cell, T cell, monocyte, granulocyte) and/or breast luminal epithelium, colon epithelium, lung alveolar epithelium, neuron, oligodendrocyte, and endothelium, we divided the genome into segments with lengths of 10–2000 bp, and then identified markers with parameters “- -min_cpg 5 - -min_cov 10 - -delta_means .3 - -pval .05.” The top 30 markers were used for further analyses. CpGs from those WGBS cell type references were filtered by intersection with CCGG flanks to align with FLEXseq’s coverage before finding markers.

#### Identification of differentially methylated regions (DMR)

We compared the methylation patterns to identify DMRs in promoter regions between six metastatic LUADs (tumor purity ≥ 60% from CNA analyses) and six negative controls of CSF. The wgbs_tools function “find_markers” (parameters “- -min_cpg 3 - -min_cov 8 - -delta_means .3 - -pval .05”) was used, with a length range of 10–1500 bp. From the initial list of regions representing cell type-specific markers, we then selected markers with the following criteria: (i) the average methylation ≥ 0.66 of one cell type and < 0.33 in all others, or vice versa, and (ii) coverage of 20x or more (across all reads that cover CpGs of the marker). We extracted the top 100 markers with the highest delta of beta values. Hypomethylated and hypermethylated DMRs were defined in the same way as the cell type-specific markers.

### Deconvolution classification

First, fragment-level deconvolution was used to estimate cell type proportions for each sample with 22 possible cell type references (see Supplementary Methods).

Next, we normalized every cell type for each sample as indicated by a *z*-score. The *z*-score measures the difference in cell type proportions between target tumors and the reference population. The reference population was defined as either the negative controls or the non-target tumors. The negative control CSF came from patients with autoimmune diseases, infections, or inflammatory conditions (without organ transplants). The *z*-score was calculated using the statistical equation *z*-score = (x – μ) / σ. We defined *x* as the cell-type proportion from the case and μ and σ as the mean and standard deviation of the cell-type proportion in the reference population, respectively. A *z*-score of 2 indicates a value of two standard deviations from the mean.

Finally, we required the top-ranked cell types to have a *z*-score > 2 and to be associated with a tumor’s COO (excluding background cell types such as smooth muscle cells and endothelium). The sample was categorized as (i) “Matched” when the qualified top-ranked cell type matched the gold standard, (ii) “Misleading profile” if it matched a different tumor type, or (iii) “Indeterminate” if there was no qualifying top-ranked cell type or if the leading cell type was oligodendrocyte. While oligodendrocytes and neurons had similar neuronal lineages as primary CNS tumors, we used neurons as the COO of CNS tumors. Samples were labeled as “Reference” when used for normalization and “N/a” (not applicable) when excluded for deconvolution classification.

### Statistical analysis

Wilcoxon rank-sum test was used to test the distribution differences of continuous variables between groups. Adjusted *P* value was used for multiple comparisons with Bonferroni correction. The chi-square test was used to test the distribution differences of categorical variables. The extreme outlier was lower than the extreme lower fence [Q1 - (3 * IQR)]. The R package ‘DTComPair’ was used to compare the sensitivity of two tests. Additionally, we did not adjust for patients' age because it is strongly associated with tumor types [7]. Root-mean-square error (RMSE) was used to assess the deconvolution differences of cell type proportions by groups. Boxplots with beeswarm plots of z-scores depicted medians with central bars, and upper and lower distribution quartiles with box edges. Receiver operating characteristic (ROC) curve Analysis and the area under the ROC curve (AUC) values were used to assess the predictive performance of deconvolution classification. Data analysis and visualization were performed using R v.4.2.0. Two-sided *P* < 0.05 was considered statistically significant. *, *P* < 0.05; **, *P* < 0.01; ***, *P* < 0.001; ****, *P* < 0.0001.

## Results

### FLEXseq design and benchmark

#### FLEXseq - theoretical coverage

The need to detect and find the COO for suspected tumors motivated us to design a genome-wide methylation profiler that overlaps with cell type-specific markers [12] and references for tumor classification [7, 9]. We started by calculating the theoretical overlap of various approaches with past references (Supplementary Table S7), focusing specifically on cfDNA (∼170 bp) and fragmented FFPE tissue DNA (∼100–500 bp) in clinical specimens.

**Figure 2.**
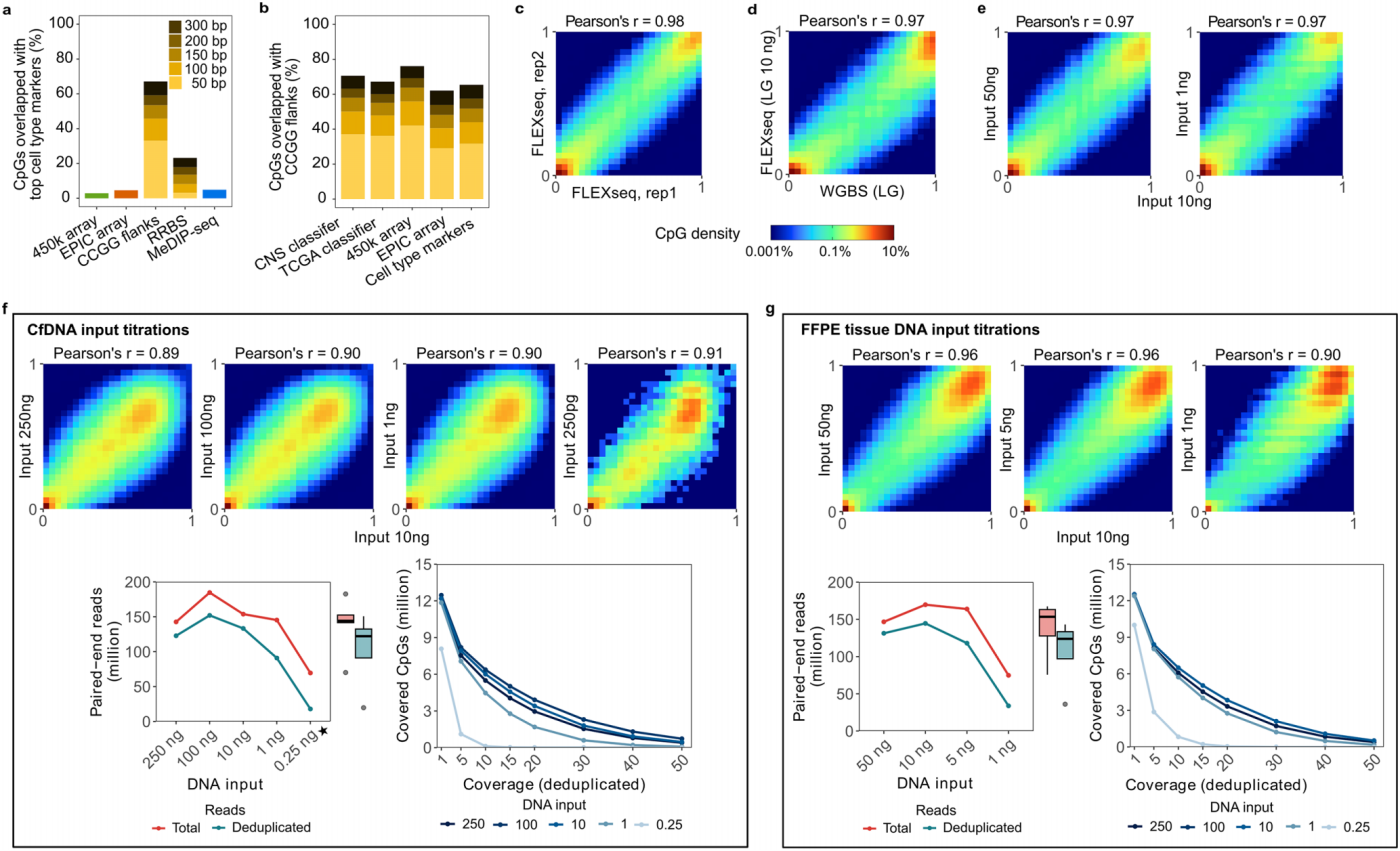
FLEXseq benchmarks. (**a**) Theoretical coverage of cell type markers (top 100 markers of each cell type) that overlap with different methods. CCGG flanks are regions flanking CCGG motifs; RRBS required two CCGG motifs set at different maximum distances and a minimum distance ≥ 30 bp; MeDIP-seq considered only hypermethylated markers identified from the WGBS data of 39 cell types from a human DNA methylation atlas (see Materials and methods). (**b**) Theoretical coverage of CpGs from CCGG flanks overlapped by different markers. The CNS classifier indicates the 32 000 most differentiated probes from the CNS tumor and control references, and the TCGA classifier indicates the 60 000 most differentiated probes from the TCGA tumor and control references. The cell type-specific markers are identified from the human DNA methylation atlas above. (**c**) Heatmap and correlation of methylation beta values between inter-run replicates (Pearson’s *r* = 0.98). (**d**) Heatmap and correlation of beta values for long-sheared (LG) K562 DNA between the gold standard WGBS and FLEXseq (Pearson’s r = 0.97). e, K562 DNA input titrations assayed by FLEXseq. Inputs were correlated with 10 ng input (Pearson’s *r* = 0.97). f, CfDNA input correlations (top), total and deduplicated unique paired-end reads (left bottom), and covered CpGs (right bottom) of a pleural fluid sample (BF3713) by FLEXseq. The dark to light blue lines indicate DNA inputs (ng). g, DNA input correlations (top), paired-end reads (left bottom), and covered CpGs (right bottom) of an FFPE tissue sample (TF640) by FLEXseq (Pearson’s *r* ≥ 0.90).

An *in silico* analysis showed that the CCGG cut sites with a flanking window of 100 bp in each direction (defined as CCGG flanks) cover 9M CpGs with the highest yield and 46% of CpGs in cell type-specific markers (Fig. 2a, Supplementary Fig. S1a–c, and Supplementary Table S8). In contrast, other methylation profiling methods, including methylation microarrays, RRBS [14, 21–23], and methylated DNA immunoprecipitation sequencing (MeDIP-Seq) [24] cover 3%-8% of those CpGs [12]. CCGG flanks also cover 36–37% of CpGs from the machine learning classifiers based on methylation array data (central nervous system [CNS] or TCGA panels) [7, 9] (Fig. 2b, see Materials and Methods and Supplementary Methods). RRBS targets CCGG flanks but effectively requires two MspI-cut ends on the same molecule, which is uncommon in fragmented DNA (0.7% genome maximal coverage for 100–bp fragments), whereas capturing fragments with at least one cut end yields 10.8% coverage and includes CpGs across a broader range of regulatory and non-canonical contexts, including enhancers, CpG shores/shelves, open sea, and intronic regions (Supplementary Fig. S1d).

#### FLEXseq - design

We designed FLEXseq to enrich CpGs in fragments with one single MspI-cut end, output accurate methylation data, and remain compatible with highly fragmented DNA input. FLEXseq involves two ligations and a double-stranded cut between the ligations, followed by methylation conversion (Fig. 1a). In the critical first step, both sides of input DNA molecules ligate to a semi-permissive adapter (Adapter A) that will serve as a blocker for untargeted background molecules and a primer landing site for targeted molecules. We then used the MspI nuclease to make double-stranded cuts, creating new and unblocked DNA ends. After an end repair, these newly exposed ends at the cut site are targeted through a second ligation with a second adapter (Adapter B) that is also needed for sequencing library formation. Because each of the two adapters at opposite ends has a necessary primer landing site (i.e. A + B are both needed), only targeted and cut DNA molecules are eventually sequenced while non-target DNA is ignored. Sequencing excludes non-target DNA with two A adapters or two B adapters. All DNA molecules have a free end associated with Adapter A that allows PCR duplicate removal and the potential of fragmentomics involving single-end motifs [25–28]. We then used enzymatic conversion [29] instead of bisulfite conversion to avoid breaking the adapter-ligated molecules. Conversion is followed by amplification and sequencing of the target molecules. Further details are in the Materials and Methods section under FLEXseq.

#### FLEXseq - analytical performance

To assess reproducibility and bias in the methylation output, we correlated K562 (Supplementary Fig. S2a) replicates and compared them with WGBS as the gold standard. Three inter-run replicates shared similar coverage of the targeted regions and were highly correlated (Pearson’s *r* = 0.98; Fig. 2c, Supplementary Fig. S2b, c, and Supplementary Table S1b), demonstrating reproducibility. FLEXseq was highly correlated with WGBS, demonstrating biological fidelity (Pearson’s *r* = 0.97 for long-sheared DNA and 0.96 for short-sheared DNA; Fig. 2d and Supplementary Fig. S2d). While FLEXseq, RRBS, and XRBS had comparable overall correlations with WGBS (Supplementary Fig. S2e), FLEXseq correlated well with WGBS for methylation beta values ranging from 0.2 to 0.8. Our reproductions of WGBS, RRBS, and XRBS correlated with public datasets (Pearson’s *r* ≥ 0.91; Supplementary Fig. S2f).

When testing against K562 DNA, >78% of FLEXseq reads aligned across all DNA lengths (Supplementary Fig. S2g). In contrast, XRBS produced < 27% usable reads from short-sheared long-sheared DNA, with the remaining reads dominated by adapter/primer dimers and short unmappable inserts. The yield improved to 71% when using libraries prepared from (original/unsheared) DNA (Supplementary Table S1a), which indicates that XRBS performance was strongly input-dependent. RRBS also performed sub-optimally with 58% alignment of unsheared K562 DNA (Supplementary Table S1a).

We assessed the DNA input limit of detection by titrating K562 DNA, cfDNA from one pleural fluid sample (BF3713), and DNA from one FFPE tissue sample (TF640). CpG beta values from 50 ng and 1 ng K562 DNA were highly correlated to 10 ng input and had similar coverage (Pearson’s *r* = 0.97; Fig. 2e). Further cfDNA dilutions down to 250 pg decreased CpG coverage, but bias in methylation remained low (Pearson’s *r* ≥ 0.90; Fig. 2f). Similarly, there was a high correlation between 1 and 50 ng of FFPE tissue DNA (Pearson’s *r* ≥ 0.90; Fig. 2g), though the number of covered CpGs decreased from 5M with 10 ng to 0.2M with 1 ng when filtering by a coverage of 15x.

The median on-target rate of K562 DNA input titrations was 96% (IQR 94–96%; Supplementary Table S1a) based on reads starting with C/TGG. Each on-target read is guaranteed to contain methylation data based on the first position. We further demonstrated that the high on-target rate was not limited to MspI by attempting FLEXseq with the TaqI-V2 enzyme that cuts at the TCGA motif. The resulting on-target rate ranged 93–95% across four samples (Supplementary Results).

**Figure 3.**
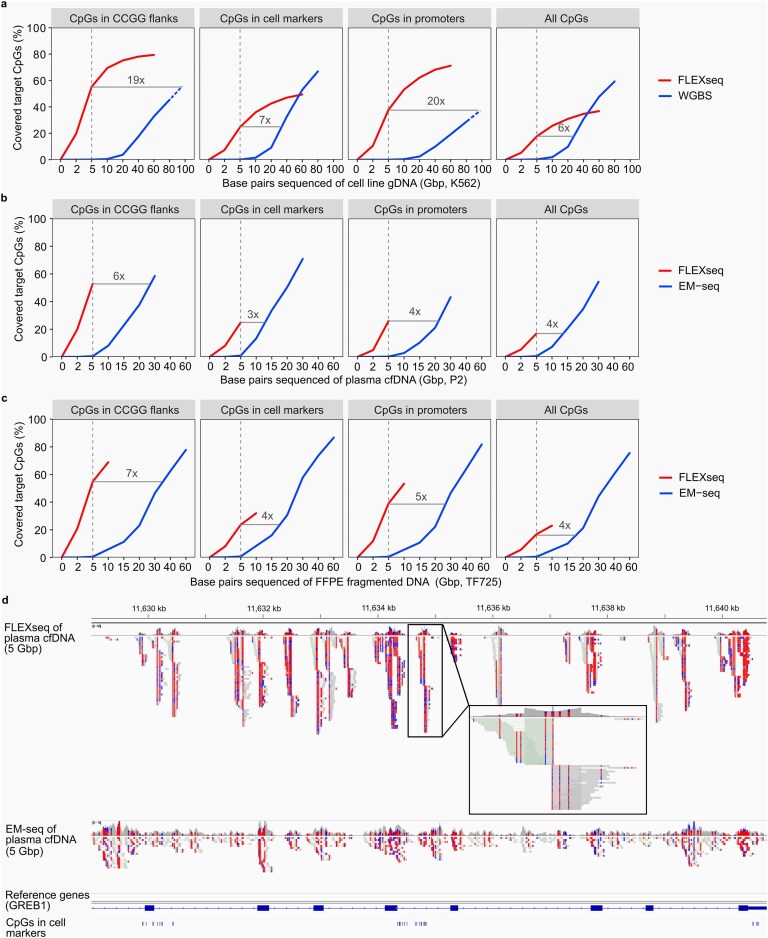
FLEXseq enrichment improves coverage. (**a, b, c**), Enrichment of CpGs in the CCGG flanking 50 bp regions, top 250 cell type-specific markers across different cell types[12], promoters, and all CpGs with coverage $ \ge $10x of FLEXseq (red) and whole-genome methylome sequencing (bisulfite conversion [WGBS] of long-sheared K562 DNA, or enzymatic conversion [EM-seq] of plasma cfDNA and fragmented FFPE tissue DNA; blue). Fold enrichment is based on 5 Gbp of two approaches. (**d**), A representative area compares equal base pairs (5 Gbp after deduplication) between WGBS/EM-seq and FLEXseq. Unmethylated (blue) and methylated (red) CpGs are highlighted. Gene and cell-type marker tracks are shown at the bottom.

To achieve the same CpG coverage of 10x in K562 long-sheared DNA, WGBS would require 19-, 7-, 20-, and 6-fold more sequencing than FLEXseq across CCGG flanks, cell type markers, promoters, and all CpGs, respectively (Fig. 3a). In plasma cfDNA (P2) or fragmented FFPE DNA (TF725), whole genome methylome sequencing with enzymatic conversion (EM-seq) similarly showed lower efficiency, requiring three- to seven-fold more sequencing than FLEXseq (Fig. 3b–c). Consistent with these CpG site-level estimates, base-level enrichment analysis showed that, among bases aligning to the targeted regions, FLEXseq yielded three-fold higher CG bases across cell-type markers and six-fold higher CG bases across promoters than EM-seq, with correspondingly denser coverage around MspI cut sites (Fig. 3d). In K562, FLEXseq also outperformed WGBS by 6-fold across cell-type markers and 16-fold across promoters, respectively, indicating better allocation of sequencing depth to informative CpGs.

### Copy number detection

**Figure 4.**
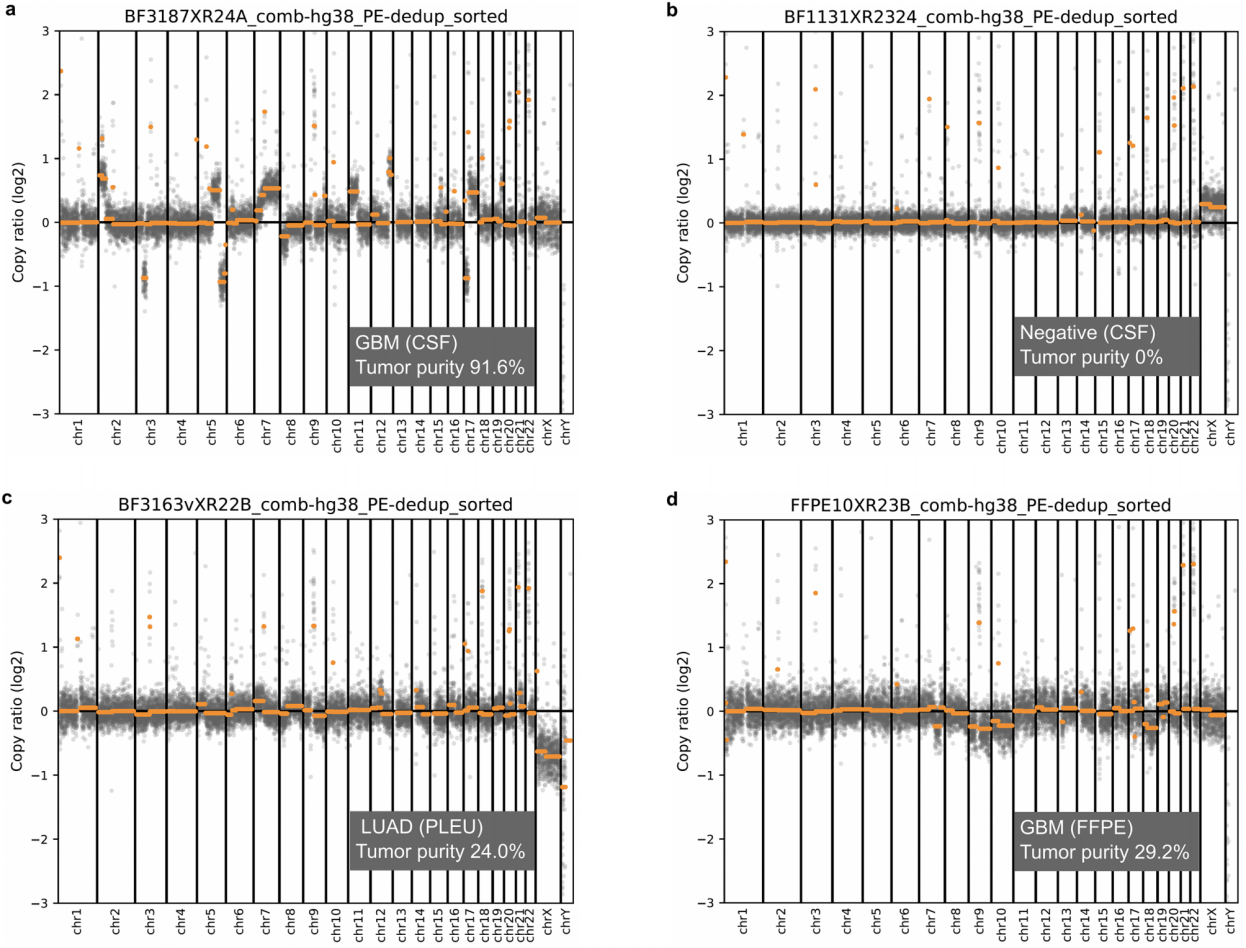
Copy number analyses from FLEXseq. Representative examples of genome-wide copy number profile changes in CSF, pleural fluid, and FFPE samples (sex chromosomes, chrX and chrY, were not adjusted). Two CSF cfDNA samples (BF3187 in (**a**) and BF1131 in (**b**), one pleural fluid sample (BF3163 in (**c**), and one FFPE tissue DNA sample (TF640 in (**d**) with estimated tumor purities were shown. The full set of plots is available (see Data Availability).

FLEXseq provided chromosomal copy number output after normalizing against control diploid references from four non-cancer CSF cfDNA samples. We detected tumor DNA by interpreting deviations from diploid as Copy Number Aberrations (CNA) and indicators of clonal aneuploidy. We also estimated tumor purity based on the difference between the log2 ratios of chromosomal segments and the baseline at gains and losses, as previously described [16, 17]. The genome-wide copy number profiles were unbalanced and aberrant as shown in representative cases (Fig. 4) and across nearly all cancer specimens (see Data Availability for plots). Additionally, we found that FLEXseq and paired whole-genome sequencing (WGS) results were comparable, except in one case (Supplementary Fig. S3).

### DMR identification

See the results in Supplementary Results for details.

### Cell-type deconvolution

#### Deconvolution of *in silico* titrations

To assess the performance of deconvolution at CCGG flanks *in silico*, we subsampled WGBS data [12] from purified cell types and intersected them with CCGG flanks to create seven titrations (Supplementary Table S3). We trialed two deconvolution approaches to predict cell type proportions for each titration: (i) CelFiE CpG-level deconvolution [30] using the top 30 cell type-specific markers (Supplementary Table S5) that excluded the titration data (Supplementary Table S4), and (ii) UXM fragment-level deconvolution [12] using the top 250 markers (Supplementary Table S2). Both methods yielded similar predicted and actual proportions (root-mean-square error [RMSE] ≤0.01; Supplementary Fig. S4a), with UXM fragment-level deconvolution remaining accurate down to 0.3% purity of target cell types.

#### Deconvolution of DNA titrations and plasma

We continued with physical titrations by mixing DNA from purified primary immune cell types (Supplementary Table S6). Fragment-level deconvolution detected target cell types down to 1% purity, and we used this approach for all further deconvolutions (Supplementary Fig. S4b and Supplementary Table S9). We then deconvoluted the titrations of DNA from cancer cell lines into DNA from immune cell mixtures (Supplementary Table S6). We found that the deconvolution underestimated the tumor cell type proportion, but the rank order of the proportions was reliable (Supplementary Fig. S4c). We then deconvoluted plasma cfDNA from healthy donors [31] using WGBS and FLEXseq data (Supplementary Fig. S4d). Cell-type proportions were similar between WGBS and WGBS intersected with CCGG flanks (RMSE = 0.02).

#### Deconvolution of clinical samples

**Figure 5.**
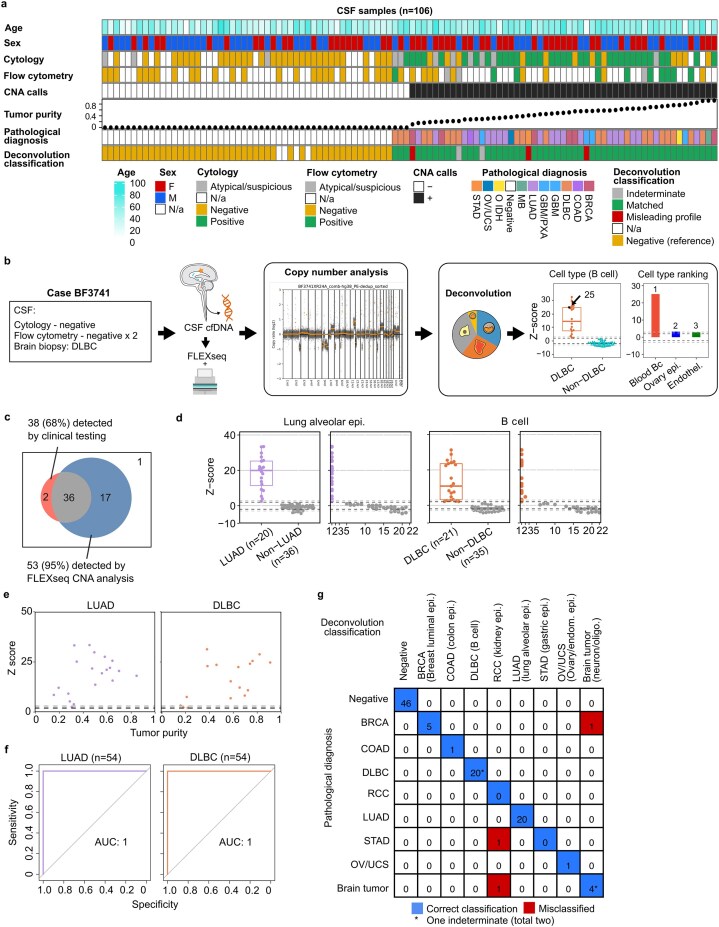
Cell-of-origin classification of CSF cfDNA based on deconvoluted cell type proportions. (**a**) A summary of demographic and clinical characteristics, copy number analysis, and deconvolution of 106 CSF cfDNA samples by FLEXseq. Four negative controls with extreme proportions were excluded. (**b**) Schematic of the workflow using a representative CSF case (BF3741) that leads to copy number and deconvolution analyses. (**c**) Tumors detected by CNA analysis using FLEXseq data compared to cytology and/or flow cytometry results. (**d**) *z*-scores of the cell-of-origin of two common CNS metastases: lung alveolar epithelium for lung adenocarcinoma (LUADs, purple) versus 36 non-LUADs (gray), and B cell for diffuse large B-cell lymphoma (DLBCs, orange) versus 35 non-DLBCs (gray). The *x*-axis in the scatter plots indicates the *z*-score rankings of the target and non-target tumors. The black and gray dashed lines are at *z*-scores of 2 and 3, respectively. (**e**), Scatter plots between tumor purities and z-scores of CSF cfDNA of LUAD and DLBC (excluding three CNA-negatives and one indeterminate). (**f**) ROC curves for determining the cell-of-origin for LUAD and DLBC. Negative controls and two indeterminates were excluded. (**g**) Confusion matrix for all 106 CSF samples based on the deconvolution classification. The blue square indicates correct classification, and the red square indicates misclassification. * One indeterminate case was not included under each tumor type. BRCA, breast carcinoma; COAD, colorectal adenocarcinoma; RCC, renal cell carcinoma; STAD, stomach adenocarcinoma; OV, ovarian cancer; UCS, uterine carcinosarcoma; brain tumor, primary brain malignancies.

We applied FLEXseq on CSF to demonstrate tumor COO determination, building on our prior finding that tumor cfDNA is detectable even in cytology-negative cases [16]. Here, we started with 548 consecutively collected CSF samples from two institutions (Stanford and University of California, San Francisco [UCSF], of which a subset was previously reported [16] (Supplementary Fig. S5). Based on past selection criteria of cases and controls [16], we included 106 samples consisting of 56 cases and 50 negative controls (Fig. 5a and Supplementary Table S10). CSF cfDNA was sequenced at a median of 154M 2 × 50 reads (IQR 116–181M; Supplementary Table S11). The C/TGG on-target rate remained high at 98%, similar to the initial K562 benchmarks.

We built a deconvolution classifier (Fig. 5b) that normalized deconvoluted cell type proportions against negative controls (*n* = 50; Fig. 5 and Supplementary Fig. S6a) via a *z*-score. Rank-ordered *z*-scores were then used to categorize a tumor based on its COO with positive tumor contents. We used the CNA analysis to detect tumor presence and estimate the tumor purity. CNA analysis from the FLEXseq data detected 95% of the CSF tumor cases, higher than the clinical testing of cytology and/or flow cytometry (68%, *P* < 0.001; Fig. 5c). The 22 cell type references used for deconvolution were from common tumor types (see Supplementary Methods). Excluding noisy cell types unrelated to common tumors, our COO determination threshold was top-ranked z-scores above two (Fig. 5d and Supplementary Fig. S6b, c). The deconvolution classifier yielded particularly high *z*-scores for LUAD (*n* = 20, COO: lung alveolar epithelium) and DLBC (*n* = 21, COO: B cell) in the context of low background (Fig. 5d). The *z*-scores were correlated to the top-ranked cell type proportions (Fig. 5e). Alternatively, we normalized *z*-scores against non-target tumors (Supplementary Fig. S6d), but low tumor purity cases increased the background variance.

The deconvolution classifier had an overall accuracy of 97% (excluding two indeterminates). The accuracy was 100% for LUAD and DLBC in our limited set of cases (Fig. 5f). Of the three misclassified cases (Fig. 5g), BF3683 was predicted to have a renal origin by deconvolution (Supplementary Table S10). Further charting showed that this patient had a concurrent RCC not known to be in the CSF. Another case, BF3369, was also predicted to have a predominant renal origin, but the gold standard was also unclear based on unusual histology and non-matching clinical methylation classification testing of the tumor tissue. The last misclassified case, BF3647, was a breast metastasis to the brain, and deconvolution showed a high proportion of cell types from the brain.

We further applied deconvolution-based classification to FFPE tissue samples (*n* = 37; Supplementary Fig. S7). We found that LUAD and DLBC can be well separated from other non-target tumors, even when normalizing non-optimally against a set of CSF-negative controls.

## Discussion

This study introduces FLEXseq, a methylation enrichment profiling assay designed for low-input, highly fragmented clinical DNA. Across liquid biopsies and FFPE tissues, FLEXseq demonstrated significantly higher informative CpG enrichment than whole methylome approaches. FLEXseq requires 3- to 7-fold less sequencing than whole methylome approaches to cover cell type-specific markers and 4- to 20-fold less sequencing to cover critical regulatory elements like promoters. FLEXseq methylation levels are concordant with the gold standard WGBS and provide sufficient genomic coverage and sequencing depth. This data then supports copy-number analysis and genome-wide deconvolution using existing references, making FLEXseq a practical approach for scalable cfDNA/fragmented FFPE tissue DNA methylome studies.

We used TET, BGT, and APOBEC enzymatic conversions [29], but alternatives include other non-destructive conversion strategies such as TET-assisted pyridine borane sequencing (TAPS) [32], direct methylation sequencing (DM-seq) [33], or non-destructive single-enzyme methylation sequencing (SEM-seq) [34]. Bisulfite conversion is possible, albeit with DNA loss. Because the enrichment occurs before PCR, we expect FLEXseq to be compatible with direct methylation readout using nanopore sequencing [35]. Moreover, the design of FLEXseq is not limited to the MspI nuclease, which is restricted to CCGG motifs. For example, we demonstrated that TaqI-V2 can target the alternative flanking region to the TCGA motif.

There are several methylation profiling assays, each with strengths and limitations depending on the application. WGBS is the gold standard and the most comprehensive method. However, its high sequencing and computational costs bar high-depth coverage and large-scale usage. Methylation microarrays are commonly used instead and can deliver high-depth outputs across nearly 1M positions (EPIC version 2). However, the high depth is predicated on using 250 ng of DNA input per the manufacturer, and even 0.4–1M positions cover only 2–4% of all CpGs and 3–8% of cell type markers (Fig. 2a), limiting deconvolution accuracy [12]. Microarrays are also restricted to human and mouse genomes. RRBS [14], [21–23] is restricted to fragments with two MspI-cut ends, which mainly covers CpG islands but limits capture of distal regulatory elements such as enhancers when using fragmented DNA (Fig. 2a, Supplementary Fig. S1). XRBS [15] has similar coverage as FLEXseq, but is practically incompatible with fragmented DNA such as cfDNA (Supplementary Fig. S2g). MeDIP-seq excels at enriching methylated cytosines but imprecisely detects hypomethylated markers, encompassing 98% of cell type-specific markers (Supplementary Table S8).

As a cost-efficient yet broad profiling technology, FLEXseq opens future possibilities. This study’s 160M reads amount to ∼$114 per sample (NovaSeqX 25B kit). By preserving one free end on every DNA molecule, it has the potential to be utilized in fragmentomics [25–28]. FLEXseq captures half of the known methylation aging markers associated with epigenetic clocks [10], [36]. Although not explored here, FLEXseq can potentially profile methylomes in non-human organisms without further development [36].

FLEXseq has some limitations. Compared to WGBS, FLEXseq does not cover all genomic loci and, therefore, is less suitable for generating comprehensive long-term reference methylomes. Likewise, as an MspI-anchored profiling assay, FLEXseq does not achieve the same depth as targeted panels using the same amount of sequencing. However, this limitation is less critical in specimens with low DNA input, such as CSF or urine, where the scarce available DNA molecules physically limit achieving high depth. In such cases, the priority shifts to integrating as many methylome markers as possible.

The CSF cases and controls were constrained to an unblinded training set with limited tumor types. For other specimens and tumors, only a series of cases was provided to demonstrate the feasibility of using FLEXseq. Future studies are needed to determine accuracy and utility under different contexts. The deconvolution classification is currently limited to 40 pure cell-type references, whereas RNA cell atlases show that > 400 cell types exist [12, 37]. Nonetheless, we expect this barrier to diminish as purified cell type references expand over time, especially with the advent of single-cell sequencing [38] and pseudo-bulk data.

In conclusion, FLEXseq is a versatile methylome profiler at single-nucleotide resolution that accelerates with low-input, fragmented DNA. It provides an accurate and cost-effective alternative to whole-genome methylome sequencing with substantial genomic coverage and high depth. Coupled with methylation deconvolution, FLEXseq enables tumor detection and classification even at low tumor purities.

## Supplementary Material

gkag385_Supplemental_Files

## Data Availability

Raw sequencing data are available for all cell lines and samples from healthy donors who gave informed consent for genomic data sharing. FASTQ files have been deposited with SRA under Bioproject PRJNA1125505. DNA methylation data across all samples are available in deidentified PAT files to preserve fragment-level read information while protecting patients′ privacy and confidentiality. PAT files, processed methylation data, copy number plots, t-Distributed Stochastic Neighbor Embedding (t-SNE) plots, and data for figures are available under Zenodo at https://zenodo.org/doi/10.5281/zenodo.19515067 and https://zenodo.org/doi/10.5281/zenodo.18880741.

## Notes

Present address: Oregon Health & Science University, Portland, OR 97239, United States

Present address: School of Medicine, University of Calgary, Calgary, AB T2N 4N1, Canada

Present address: Barts Cancer Institute, Queen Mary University of London, London EC1M 6AU, United Kingdom

Present address: Centre for Epigenetics, Queen Mary University of London, London EC1M 6AU, United Kingdom
