# Supplementary material for: Enriched methylomes of low-input and fragmented DNA using fragment ligation EXclusive methylation sequencing
Source: Nucleic Acids Res. 2026 May 14;54(9):gkag385. doi: 10.1093/nar/gkag385 (PMC13171454; doi:10.1093/nar/gkag385)
Supplement: gkag385_Supplemental_Files [file gkag385_supplemental_files.zip › 26-0304-MethodsPaper_SupplementaryData_clean.docx]

**Title: Enriched Methylomes of Low-input and Fragmented DNA Using Fragment Ligation EXclusive Methylation Sequencing**

Jingru Yu^1^, Lauren S. Ahmann^1^, Yvette Y. Yao^1^, Angus Toland^1^, Alicia Snowden^2^, Chandler Ho^3^, Netanel Loyfer^4^, Tommy Kaplan^4,5^, Hannes Vogel^1,6^, Linlin Wang^7^, Brooke E. Howitt^1^, Brittany Holmes^1^, Alarice Cheng-Yi Lowe^1^ and Wei Gu^1^*

^1^ Department of Pathology, School of Medicine, Stanford University, Stanford, CA 94305, USA

^2^ Howard University College of Medicine, Washington, DC 20059, USA

^3^ Clinical Laboratories, Stanford Health Care, Stanford, CA 94305, USA

^4^ School of Computer Science and Engineering, The Hebrew University of Jerusalem, Jerusalem 9190501, Israel

^5^ Faculty of Medicine, The Hebrew University of Jerusalem, Jerusalem 9112001, Israel

^6^ Department of Pediatrics, School of Medicine, Stanford University, Stanford, CA 94305, USA

^7^ Department of Laboratory Medicine, School of Medicine, University of California San Francisco, San Francisco, CA 94143, USA

* To whom correspondence should be addressed. Email: weigu@stanford.edu

Present Address:

Lauren S. Ahmann, Oregon Health & Science University, Portland, OR 97239, USA

Yvette Y. Yao, School of Medicine, University of Calgary, Calgary, AB T2N 4N1, Canada

Angus Toland, Children’s Hospital Colorado, University of Colorado Anschutz Medical Campus, Aurora, CO 80045, USA

**Supplementary Data**

1. **Supplementary Methods**
2. **Supplementary Results**

Specimen QC and characteristics

FLEXseq targeting the TCGA motif with TaqI-V2

Identification of differentially methylated regions (DMR)

1. **Supplementary Figures**
2. **Supplementary Tables**

**Tables in the supplementary .xlsx file:**

Table S1a. Quality control metrics of FLEXseq of benchmark samples

Table S1b. Correlations between different methods

Table S1c. Coverages and depths of subsampled K562 benchmark samples from FLEXseq, RRBS, and XRBS

Table S2. UXM fragment-level deconvolution markers

Table S3. Cell type proportions of *in silico* samples from the brain, lung, liver, and colon

Table S4. CpG-level deconvolution references for *in silico* samples

Table S5. CpG-level deconvolution markers for *in silico* samples

Table S6. DNA titrations of four immune cell types and tumor cell lines mixed with the DNA mixtures of four immune cell types

Table S7. *In silico* coverage of RRBS, XRBS, FLEXseq, methylation arrays, and MeDIP-seq

Table S8. Methylation status of the top 100 cell type-specific markers across 39 cell types from a human DNA methylation atlas

Table S9. CpG-level deconvolution markers for DNA titrations of four immune cells

Table S10. Demographic, clinical information and classifications of patients used in this study

Table S11. Quality control metrics of FLEXseq samples

1. **Supplementary Protocol**

**Supplementary Methods**

**Sample collection**

We used CSF and non-CSF body fluid samples derived from previous studies(1–3) and new collections. New body fluid samples were consecutively from Cytology and Flow Cytometry Laboratories at Stanford Healthcare between 2020 and 2023. Specimens from cytology were spun following the clinical standard operating procedure (~800-1600 x g for 10 minutes or by gravitational settling). The supernatant was then pipetted or decanted and then stored at refrigeration until it was processed for cfDNA. Body fluids specimens from flow cytometry were kept at 4 °C for up to 4 weeks and then processed. For fine needle aspirates (FNA), the clinical protocol used normal saline fluid from needle rinses or specimens placed directly into normal saline. In addition, we included an ovarian cyst fluid (BF3526) from the Stanford Tissue Bank. During specimen processing, all original or supernatant fluid samples were centrifuged at 16,000 x g for 10 minutes, and the hard-spun supernatant was stored at -80 °C.

**Clinical specimen selection criteria**

Based on our previous studies(1, 2), CSF cases and controls were defined below. Negative controls (NegCtrl, e.g., infection, neuroinflammatory, or autoimmune disease) were patients with at least six months of cancer-free follow-up and no cancer history in the past five years. Positive cases (CytoFlowPos) included patients with primary or metastatic brain malignancies who had positive results in cytology or flow cytometry, excluding those with atypical or suspicious findings. Additionally, patients with a probable positive testing result (ProbPos) were initially screened using WGS as previously described. Those with positive CNA detection were also qualified as cases. The inclusion criteria of ProbPos are one of the following: i) a positive cytology or flow cytometry result of the body fluid from different time points, ii) a biopsy diagnosis, iii) unequivocal imaging findings, iv) atypical or suspicious cytology results, or v) a diagnostic consensus to treat. Samples with a diagnosis of leukemia and T-cell lymphoma were excluded.

We selected 37 cases with FFPE tissue DNA from patients with a clear pathological diagnosis. The tissues were across various body sites to check the feasibility of FLEXseq. Among those samples, 35 of them had high histological tumor purity > 50%.

We assayed non-CSF cfDNA from a variety of body fluids with CytoFlowPos or CNA-positive ProbPos, including pleural fluid (n = 20), FNA supernatant (n = 10), abdominal/peritoneal/ascitic fluid (n = 10), pelvic wash fluid (n = 1), and ovarian cyst fluid (n = 1).

The clinical and demographic information of all patients was reviewed retrospectively from medical records. Patients in the CSF case group (median 59.5, IQR 52.8-67.0) were older than the negative controls (median 41.0, IQR 31.8-60.0, *P* < 0.001) and similar by sex (*P* = 0.122). Pathological diagnosis serves as the gold standard for tumor classification. For patients with multiple time-point samples, only the earliest sample was used.

**Calculation of DNA input**

To calculate the DNA input amount of each well, a titration curve was generated by spiking a constant amount of lambda phage DNA (10 pg) into normal plasma (P2) titrated to 1ng, 0.5ng, and 0.25ng. Because the constant amount of lambda DNA was also spiked into each sample well, the ratio of human reads (total reads - phage reads) to lambda reads against the known starting concentrations can be used to calculate the total amount of DNA per well. The equation below was used to calculate sample DNA concentrations (ng/mL). Each prepared sample was split into two before amplification to minimize PCR duplicates. When calculating the concentration, the DNA of each split well obtained from the calibrated titration curve was added together to represent the total starting DNA amount. For samples with multiple split wells, the concentration was calculated separately for each run and then averaged across all runs. The qPCR data from the same sample were analyzed separately.

Equation

$$Sample DNA concentration \left( \frac{ng}{mL} \right)$$

$=(\frac{ng per well}{Volume of DNA into library prep (uL)})\times(\frac{Eluted volume from extraction (uL)}{mL sample extracted})$ (1)

**Obtaining and processing external data**

Outside data was obtained and pre-processed as follows: We downloaded the WGBS data for K562 cells (SRR4235743) as raw FASTQ files. The paired-end WGBS data was trimmed by cutadapt v.4.4 function with parameters ‘-a AGATCGGAAGAGC -A AGATCGGAAGAGC’. Ten bp were ignored at both the 5' and 3' end of Read 1 and Read 2 during the methylation calling process, to reduce the random priming bias. Other pre-processing steps were similar to FLEXseq. We also downloaded the RRBS data (GSM683856 and GSM683780) as raw FASTQ files. The single-end RRBS data was trimmed using trim_galore function from TrimGalore v.0.6.10, with parameters ‘--rrbs --phred64 --quality 33 --illumina’. Other pre-processing steps were similar to FLEXseq with single-end data. XRBS data (GSM4518657 and GSM4518658) derived from 10 ng DNA were downloaded as methylation metadata.

WGBS data of plasma cfDNA from 39 healthy individuals was obtained from Gao and colleagues(4) (CRA001142). The raw FASTQ files were processed following a similar pipeline as FLEXseq but with a trimming with parameters ‘-u 10 -U 10’ and ‘-u -10 -U -10’. We used the bismark_methylation_extractor function to get methylation calls for individual CpGs. Covered CpG sites were further intersected with CCGG flanks to align with FLEXseq data. To increase the sequencing coverage, we randomly merged 13 patients to create three combined samples with a median coverage of 10X.

**Identification of microarray probes**

The raw methylation IDAT files of reference and validation samples were downloaded and processed using the minfi R package and normalized using the MNPpreprocessIllumina R package as previously described(5). They were 7,000+ solid tumor references from the TCGA datasets (TCGA, https://www.cancer.gov/tcga), 28 control references from Koelsche et al.(6), and 2,801 CNS tumor and control references from Capper et al.(7). The datasets were converted from the hg19 to hg38 reference using LiftOver. Probes in the following categories were removed: i) targeting the sex chromosomes X (chrX), Y (chrY), and the mitochondrial chromosomes (chrM) (n = 11,551); ii) containing SNPs (dbSNP153Common, n = 17,428); iii) not mapping uniquely to human reference genome hg19 (n = 3,965), and iv) not included on the EPIC array (n = 32,260). We reported the batch-adjusted analyses of CNS tumors and other tumors from the TCGA databases.

The samples from the TCGA datasets were filtered by tumor purity, based on the ABSOLUTE method (TCGA, https://www.cancer.gov/tcga) and classifier scores(5) to ensure > 7 samples are in each group with either tumor purity >50% if the reference size < 20, tumor purity > 60% if the reference size ≥ 20 and within the highest 250 scores, or tumor purity > 70% when the scores are unknown. All 2,801 CNS tumor and control samples were used to identify the most differentiated probes. At last, 60,000 and 32,000 probes from the TCGA and CNS tumor array data were used, respectively.

**Copy number analysis**

Proper normalization is critical to denoise copy number plots. We employed a cfDNA pooled reference to normalize FLEXseq regions for CNA calling. First, a healthy control plasma (P2) was used as an initial reference. We ran CNVkit on ‘wgs’ mode and used a hg38 flat file to split the genome into 200 kbp blocks. This reference was then used to generate CNR files of a randomized selection of four CSF cfDNA samples (BF3040, BF3222, BF3241, and BF3361) with no malignancies and > 50 million reads. Noise in each sample was evaluated using the CNVkit metrics command, and one sample with high-level noise (BF3014, with a larger biweight midvariance, median absolute deviation, and IQR) was removed. The final pooled reference utilizing those four BAM files from CSF cfDNA was generated following the same steps as with the P2 reference. We did not track and normalize the sex of specimens, and therefore, copy numbers from the sex chromosomes are not reliable and subsequently ignored during analyses.

We then generated log2copy ratio plots for all body fluid and FFPE samples based on the pooled reference and visualized them across all bins. The gray points in the plots are binned ratios from CNR files and the orange lines are inferred discrete copy number segments derived from CNS files. We derived the segments based on the default Circular Binary Segmentation algorithm from the DNAcopy R package. Samples were considered CNA-positive if the copy ratio plot showed one or more significant CNAs, excluding sex chromosomes.

Copy number plots of WGS data were generated using a similar process to FLEXseq data, by normalizing against WGS diploid references.

Tumor purity was estimated using the log2 copy ratio from segments larger than 10 Mbp by measuring the maximal deviation from the baseline, which was assumed to be diploid. Certain deletions or gains were assumed to be single copy changes (e.g., monosomy or trisomy). The following equation was used to determine the tumor purity as previously described(1, 8).

Equation

$Tumor Purity (\%) =\frac{1-2^{(log2 ratio)}}{1- \frac{(assumed ploidy)}{2}} \times100$ (2)

**Cell type deconvolution**

We conducted deconvolution analyses using CpG-level CelFiE (expectation-maximization algorithm)(9) and fragment-level UXM (non-negative least-squares algorithm)(10, 11). For the CpG-level deconvolution, the top 30 markers across different cell type references were identified and filtered with the criteria mentioned in the section ‘*Genomic Segmentation and Identification of Cell Type-specific Markers*’ in Materials and Methods. Methylated counts and total counts for each individual CpG within the marker were summed up to calculate the marker’s beta value.

For the fragment-level deconvolution, the top 250 unmethylated markers across different cell type references were used. Those markers were extracted directly from Loyfer et al.(10). We removed markers on the sex chromosomes and those that overlapped with common SNP positions. Different cell type references were used across various scenarios:

When deconvoluting *in silico* samples, we used 180 markers for CpG-level deconvolution and 1,500/1,750 markers (FLEXseq data covers ~60% of them) for the fragment-level deconvolution. These markers not only came from four immune cell types (B cell, T cell, mono/macrophage, and granulocyte) and endothelium, but also from the specific tissue cells. We included hepatocyte for liver mixtures, colon epithelium for colon mixtures, lung alveolar epithelium for lung mixtures, and neuron and oligodendrocyte for brain mixtures. When deconvoluting physical immune cell titrations, we used 120 (CpG-level) or 1,000 (fragment-level) markers across the four immune cell types.

When deconvoluting tumor cell line DNA titrations, we used the fragment-level deconvolution with two reference sets: i) B cell (immune/B cell lymphomas), T cell (immune/T cell lymphomas), mono/macrophage (immune), granulocyte (immune), endothelial and smooth muscle cell (vasculature), and the specific tumor cell-of-origin (luminal and basal breast epithelium for breast carcinoma, colon and small intestine epithelium for colon carcinoma, or neuron and oligodendrocyte for primary CNS tumors and neuronal cells); or ii) additional 11 cell types associated with common epithelial malignancies, including lung alveolar and bronchial epithelium (lung carcinoma, LUAD), head and neck epithelium (head and neck carcinoma, HNSC), hepatocyte (hepatocellular carcinoma, LIHC), pancreatic ductal epithelium (pancreatic carcinoma, PAAD), gallbladder epithelium (gallbladder cancer, GBC), ovarian and endometrium epithelium (ovarian and uterine carcinomas, OV/UCS), gastric epithelium (gastric carcinoma, STAD), kidney epithelium (renal cell carcinoma, RCC), and bladder epithelium (bladder carcinoma, BLCA).

When deconvoluting CSF cfDNA and FFPE tissue DNA, we used fragment-level deconvolution with those 22 references described above, which were associated with leptomeningeal spread, parenchymal brain metastasis, primary CNS tumors, and other solid tumors.

**Supplementary Results**

**Specimen QC and characteristics**

We sequenced samples with cycle threshold (Ct) > 14 from quantitative polymerase chain reaction (qPCR). To precisely quantify DNA input after sequencing, we used a lambda phage DNA spike-in across all samples and a plasma titration curve with known DNA inputs from a healthy donor (Extended Figure S1a-b). As expected, CSF cfDNA concentration (median 7.1 ng/mL, IQR 2.8-14.7 ng/mL) was lower than non-CSF body fluids (adjusted *P* = 0.008 for abdominal fluid and < 0.001 for pleural fluid, Extended Figure S1c). Cancer cases had higher DNA concentration than autoimmune/autoinflammatory cases and lower than infection cases (adjusted *P* ***=*** 0.006 and 0.04, respectively, Extended Figure S1d). One sample with incomplete methylation conversion was dropped based on an unmethylated lambda DNA non-conversion rate > 2% or a pUC-19 false positive conversion rate < 90%. Samples with < 30 million deduplicated reads were also excluded (n = 6).

**
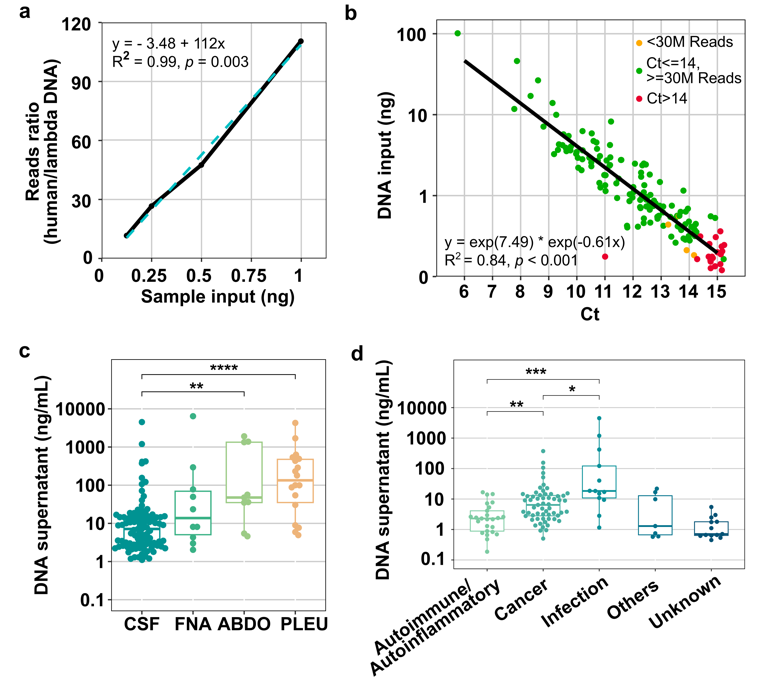
**

**Extended Figure S1:** CfDNA inputs of CSF and non-CSF body fluids. **a,** The plasma cfDNA input titration curve used to calculate sample input. The curve is based on a constant quantity of lambda phage DNA spiked into every sample before library preparation. The titration is calibrated by a linear pre-quantified healthy plasma sample (P2) (R^2^ = 0.99). **b,** The correlation between CSF cfDNA input (ng) extracted at Stanford (n = 152) and Ct from qPCR. Samples that have multiple library preparations are represented separately. Dots are shifted within ±0.25 Ct of the actual Ct value visually to see all data points without overlaps. The equation for the best fit log-linear regression (R^2^ = 0.84) is shown. The Y-axis is logged. **c,** CfDNA concentrations calculated based on the cfDNA input titration curve in (a). The 92 CSF, 10 fine needle aspiration (FNA) saline wash fluid, nine abdominal/peritoneal/ascitic fluid (ABDO), and 18 pleural fluid (PLEU) samples were shown, which were extracted at Stanford and met quality metrics (Ct ≤ 14 and ≥ 30 million deduplicated paired-end reads). CSF cfDNA concentration is lower than abdominal (adjusted *p* = 0.008) and pleural fluids (adjusted *P* < 0.001). Only significant p-values are labeled. The Y-axis is logged. **d,** CSF cfDNA concentrations of all CSF samples extracted at Stanford (n = 117, no quality metric filtering) based on diagnostic categories: autoimmune/autoinflammatory (n = 25), cancer (n = 59), infection (n = 13), other (n = 7), and unknown cause (n = 13). Inputs are highest in the infection group, followed by the cancer and autoimmune/autoinflammatory group (infection vs. cancer, adjusted *P* = 0.04; cancer vs. autoimmune/autoinflammatory, adjusted *P* = 0.006). The Y-axis is logged.

**FLEXseq targeting the TCGA motif with TaqI-V2**

To demonstrated that FLEXseq is not limited to MspI and the motif ‘CCGG’, we implemented FLEXseq with the TaqI-v2 nuclease (NEB, part number R0149) targeting the 'TCGA' motif. Cutsmart buffer was used as recommended by the manufacturer. The on-target rate was estimated by dividing reads starting with CGA or TGA by the total reads on the side cut by the nuclease. Similar to FLEXseq with MspI, the on-target read is guaranteed to contain CpG methylation data based on the first position (Extended Figure S2).


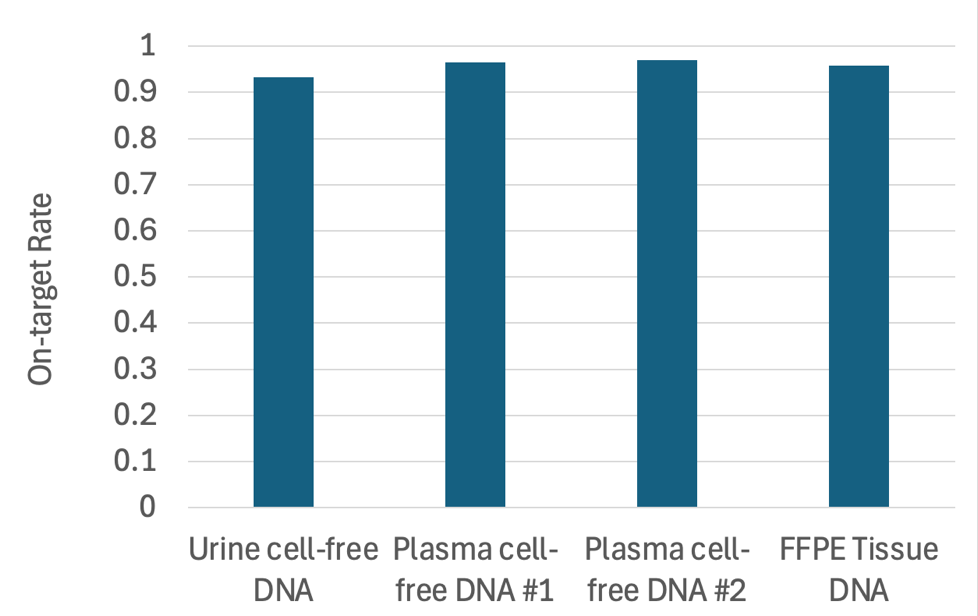


**Extended Figure S2:** On-target rate of the TCGA motif. The on-target rate of FLEX-seq using the TaqI-v2 nuclease. On-target was calculated by dividing reads starting with CGA or TGA by the total reads.

**Identification of differentially methylated regions (DMR)**

In total, 18,991 hypermethylated DMRs were identified using wgbstools (with at least three CpGs, minimum coverage of eight per CpG and 20 per region [accumulated coverage of CpGs in that region], and length between 10-1,500 bp). We found that 4,208 promoters cover those DMRs (within ±1,000 bp flanking the transcriptional start site). Of 4,208 total promoters, 1,806 cover the DMRs that are hypermethylated in metastatic LUAD, and others cover the hypomethylated DMRs. We visualized the methylation levels of those promoters in 37 samples (including 26 LUAD from CSF or pleural fluid samples, five FFPE tissues from the lung, and six negative controls from CSFs) in Extended Figure S3a.

We then identified 11 promoter regions overlapping the top 100 DMRs. Representative genes are shown in the Extended Figure S3b-e. Notably, *OXCT2* is critical for ketone metabolism, *RCSD1* regulates cytoskeletal dynamics, and *TMEM204* modulates cell adhesion and angiogenesis. Promoter hypermethylation of these genes may silence their expression, potentially contributing to cancer progression(12–14). Additionally, *HOXA9* promoters exhibited significantly greater hypermethylation in metastatic LUAD compared to controls, consistent with prior studies(15).


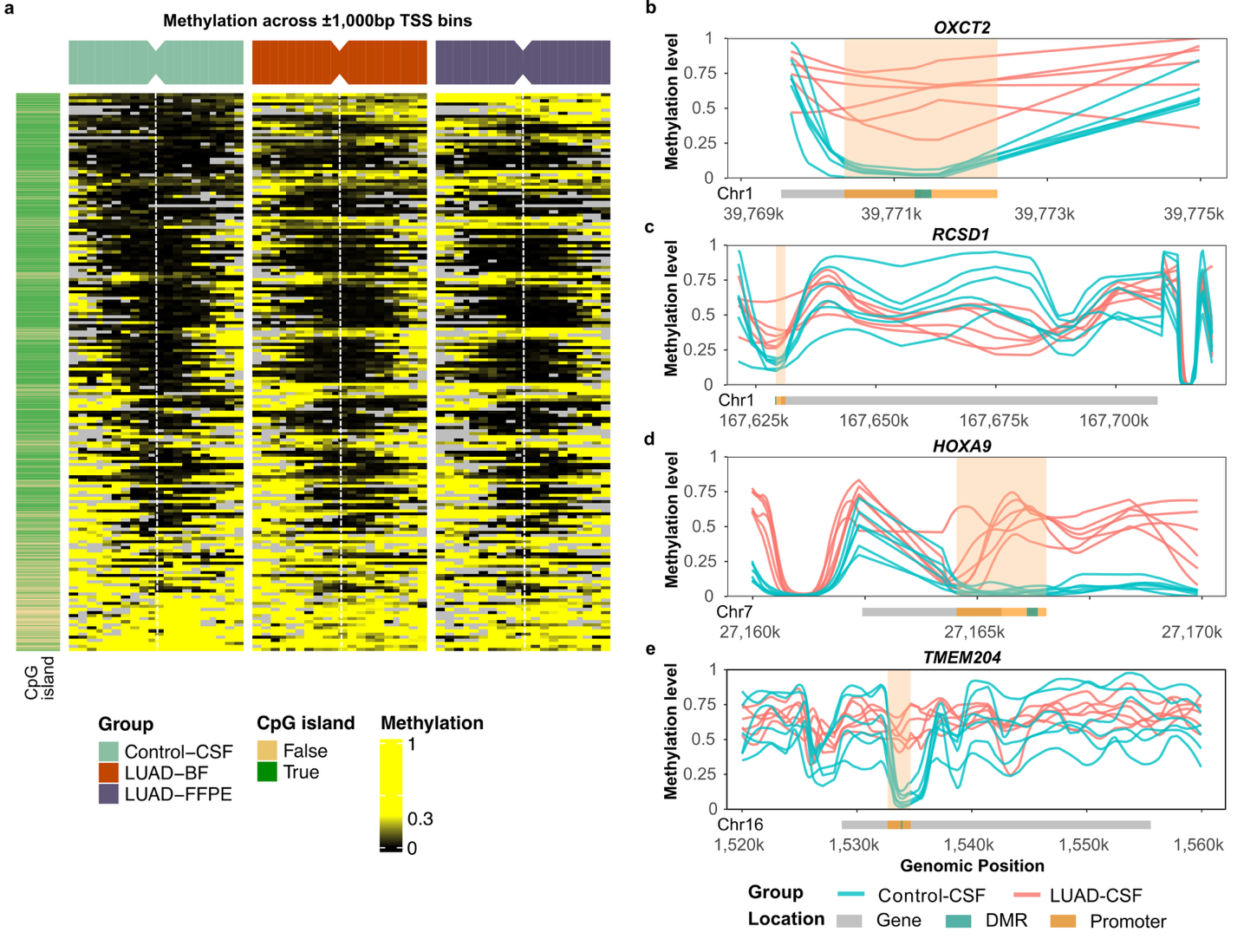


**Extended Figure S3:** Promoter DNA methylation in metastatic LUAD. **a**, A total of 4,208 gene promoters from 3,183 unique RefSeq genes harbored DNA methylation (yellow) among negative CSF controls (with infection and/or inflammation, cyan), and metastatic LUAD from CSF/pleural fluid (red) and FFPE tissue (purple) samples. Promoter methylation levels are indicated by varying shades of yellow, with each row representing a unique 100-bp promoter region flanking ±1,000 bp around the transcription start site (TSS), marked by a white dotted line. The gray bin indicates missing methylation data. Promoters with more than 30 bins (50%) of missing values were dropped. The first column highlights CpG islands (green) within gene promoters. Promoters are sorted by ascending methylation bin counts in the Control-CSF group and descending counts in the LUAD-FFPE group. **b-e**, Methylation levels in four representative genes, including *OXCT2*, *RCSD1*, *HOXA9*, and *TMEM204*. The cyan lines indicate Control-CSF samples (n = 6), and the red lines indicate LUAD-CSF samples (n = 6). The orange shade indicates the promoter location. The gray, cyan, and orange bars indicate the location of gene, differentially methylated region (DMR), and promoter, respectively.

**Supplementary Figures**

**
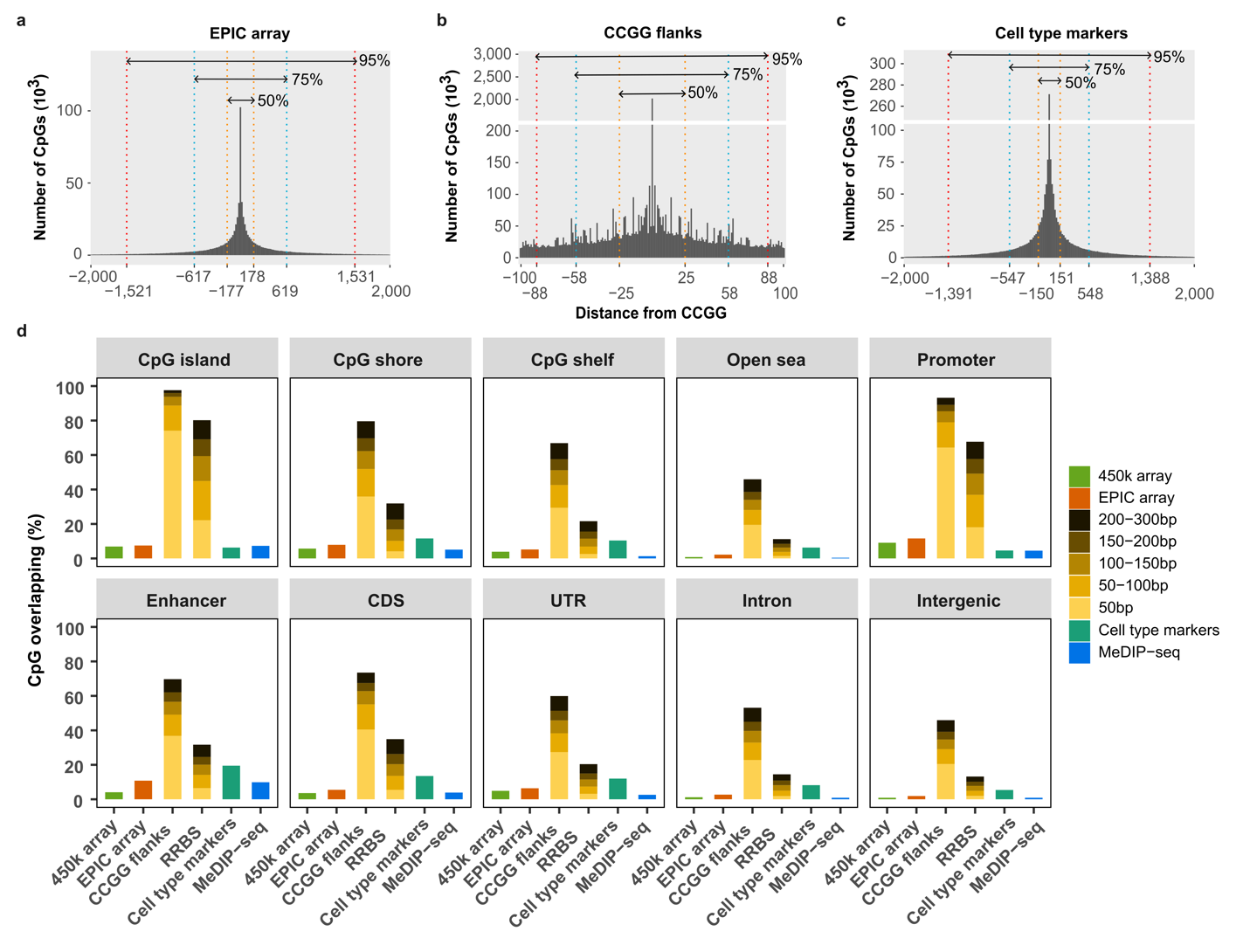
**

**Supplementary Figure S1:** *in silico* coverage of CpGs across genomic regions by different methods. **a,b,c,** Theoretical distance from the CCGG motifs for all CpGs covered by EPIC array, CCGG flanks, and cell type markers. CCGG flanks are the 50-300 bp regions flanking CCGG motifs. Cell type markers are defined across 39 purified cell types from a human DNA methylation atlas. The ranges between the orange, blue, and red dashed lines indicate the 50%, 75%, and 95% distribution of CpGs, respectively. **d**, CpG percentages across genomic regions by different methylation detection methods. RRBS regions are two CCGG motifs within a maximum distance of 50-300 bp. MeDIP-seq markers are defined as CpGs in hypermethylated regions derived from the above cell type-specific markers. CDS, coding sequence; UTR, untranslated region, including 5’ and 3’ UTR.

**
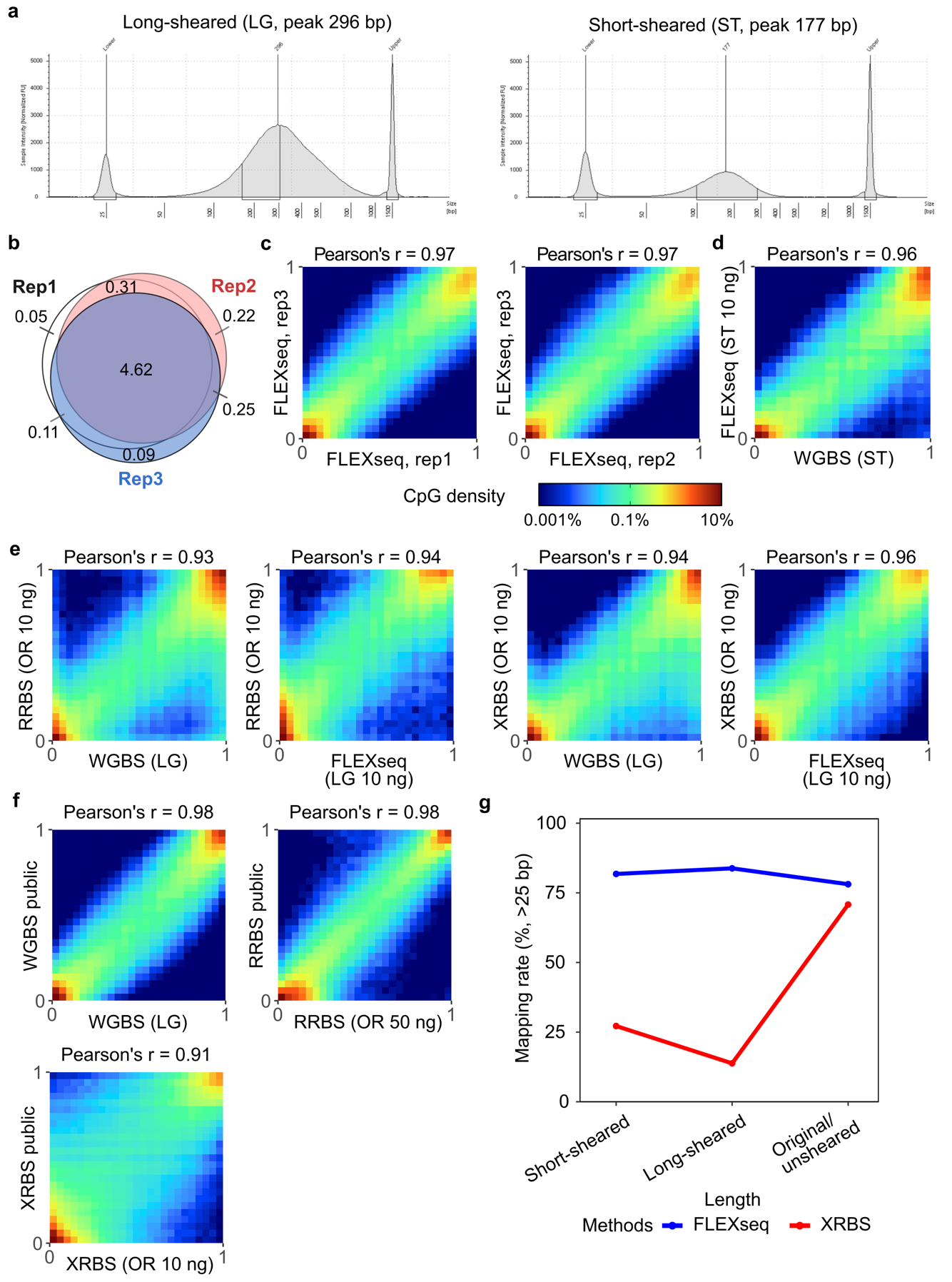
**

**Supplementary Figure S2:** K562 DNA fragment distributions and correlations between WGBS, FLEXseq, RRBS, and XRBS of K562 gDNA. All correlations are based on individual CpG methylation beta values. **a**, Fragment distributions of long- (LG, peak 296 bp) and short- (ST, peak 177 bp) sheared K562 DNA after sonication. **b**, CpG overlaps (million) between three FLEXseq replicates within CCGG motifs flanked by 50 bp with deduplicated coverage of 5x. **c**, Correlations between replicates of long-sheared K562 DNA from different batches (Pearson’s r = 0.97). **d**, Correlation of short-sheared K562 DNA between the gold standard WGBS and FLEXseq (Pearson’s r = 0.96). **e**, Correlations of K562 DNA between WGBS (LG), RRBS (optimal for original [OR] DNA, Pearson’s r = 0.95), and XRBS (more reads for OR DNA, Pearson’s r = 0.94), and between FLEXseq (LG), RRBS (OR, Pearson’s r = 0.95), and XRBS (OR, Pearson’s r = 0.96). **f**, Correlations between in-house WGBS, RRBS, and XRBS and the public data (Pearson’s r ≥ 0.91, see Supplementary Methods in detail). **g**, Percentage of mappable sequencing reads after alignment for both FLEXseq and XRBS libraries, derived from K562 DNA samples with different fragment lengths.


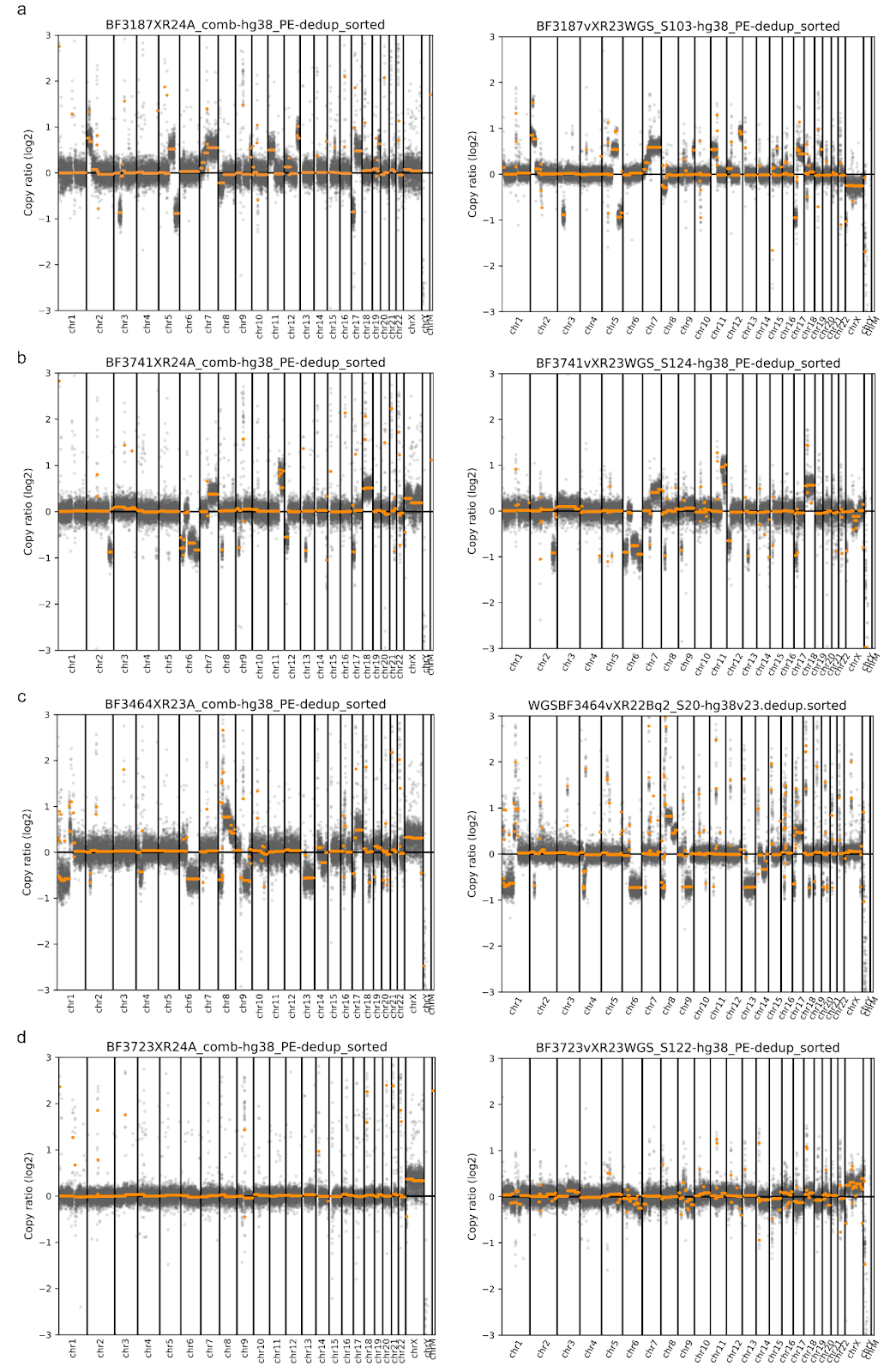


**Supplementary Figure S3:** Copy number analyses from FLEXseq and WGS. Paired copy ratio plots between FLEXseq (left) and WGS (right) are shown. Comparable results were observed across the chromosomal regions, except sex chromosomes (chrX and chrY), which were not adjusted for sex. Two tumor-positive samples, BF3187 (**a**) and BF3741 (**b**), were identified from CSF cfDNA. The third, BF3464 (**c**), was positive from cfDNA in saline wash fluid obtained during a liver fine-needle aspiration (FNA) biopsy. **d**, BF3723 was the only case overall with CNA-positive in WGS data but not from FLEXseq. The full set of plots is available (see Data Availability).


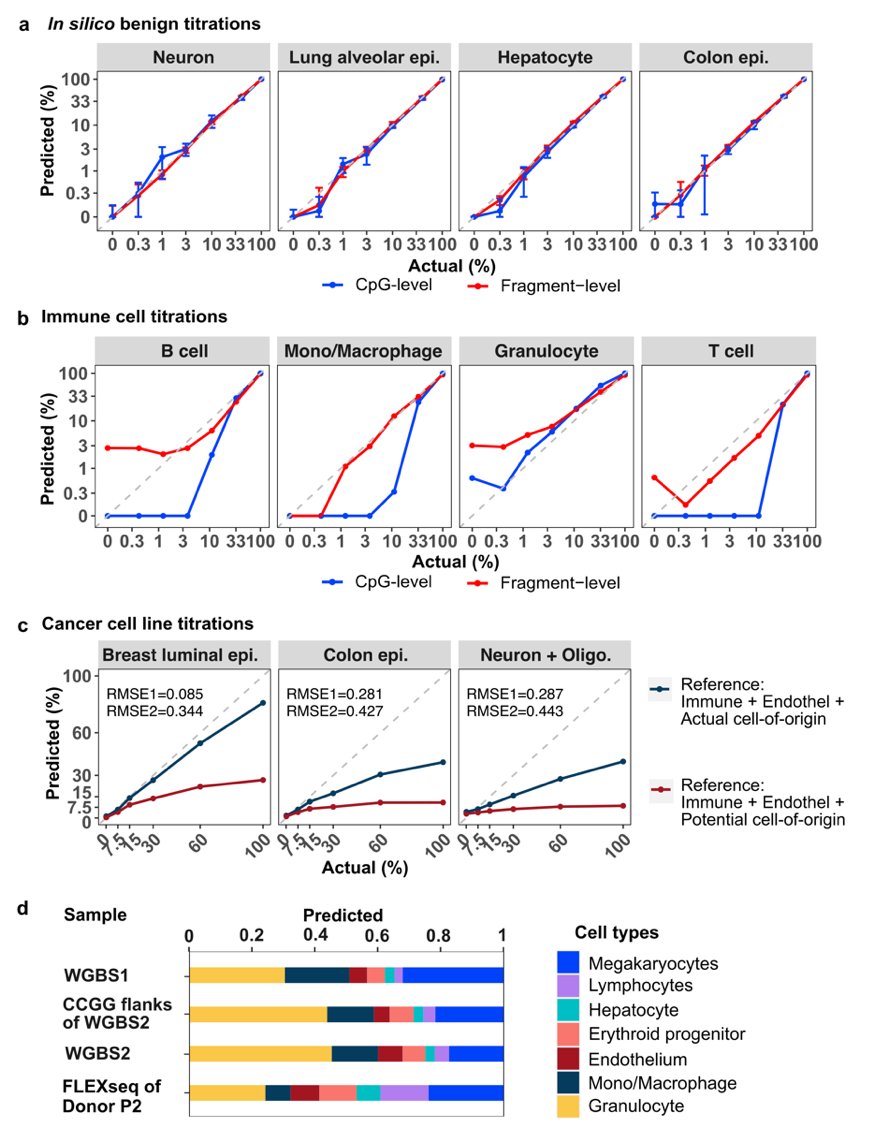


**Supplementary Figure S4:** Deconvolution of *in silico* titrations, physical DNA titrations, and plasma. **a,** Cell type proportions deconvoluted from *in silico* mixtures using the WGBS references (WGBS reads intersected with CCGG flanks). CpG- (blue) and fragment-level deconvolution methods (red) are shown. Error bars indicate the SD at each titration level. **b,** Deconvolution of gDNA of B cells, monocytes, neutrophils, and T cells titrated into mixtures of three immune cell types, respectively. **c,** Fragment-level deconvolution of BRCA (tumor cell-of-origin, breast luminal epithelium), COAD (tumor cell-of-origin, colon epithelium), and GBM (approximate tumor cell-of-origin lineage, neuron and oligodendrocyte) cell line DNA titrated into mixtures of the same four immune cell types in (a). The dark blue line with RMSE1 indicates deconvolution with references exclusive to the approximate tumor cell type and background immune cells. The dark red line with RMSE2 indicates deconvolution with a broader set of reference cell types encompassing common brain metastases. RMSE, root-mean-square deviation. **d**, Deconvolution of healthy plasma samples from Loyfer et al. (WGBS1), Gao et al. (WGBS2), WGBS2 intersected with CCGG flanks to simulate FLEXseq data, and FLEXseq from a healthy donor (P2). BRCA, breast carcinoma; COAD, colorectal adenocarcinoma; GBM, glioblastoma.


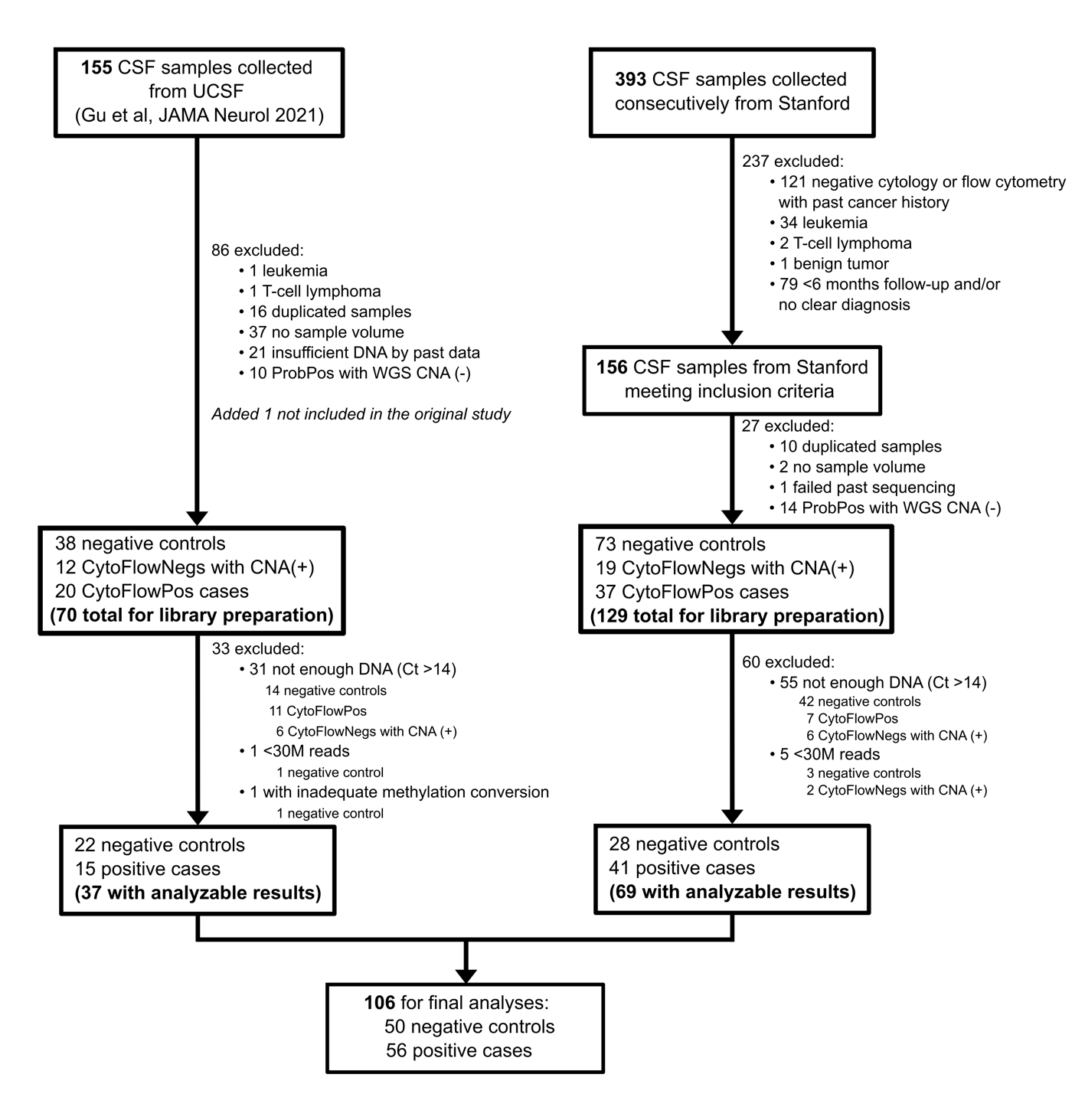


**Supplementary Figure S5:** Flowchart of CSF sample collection for the case-control study. CytoFlowPos - cytology or flow cytometry positive; CytoFlowNeg - cytology and flow cytometry negative, atypical, or suspicious; ProbPos - Negative by cytology and flow cytometry but positive based on other past criteria (see Methods); Ct - cycle threshold; WGS - whole genome sequencing; CNA - copy number aberration.


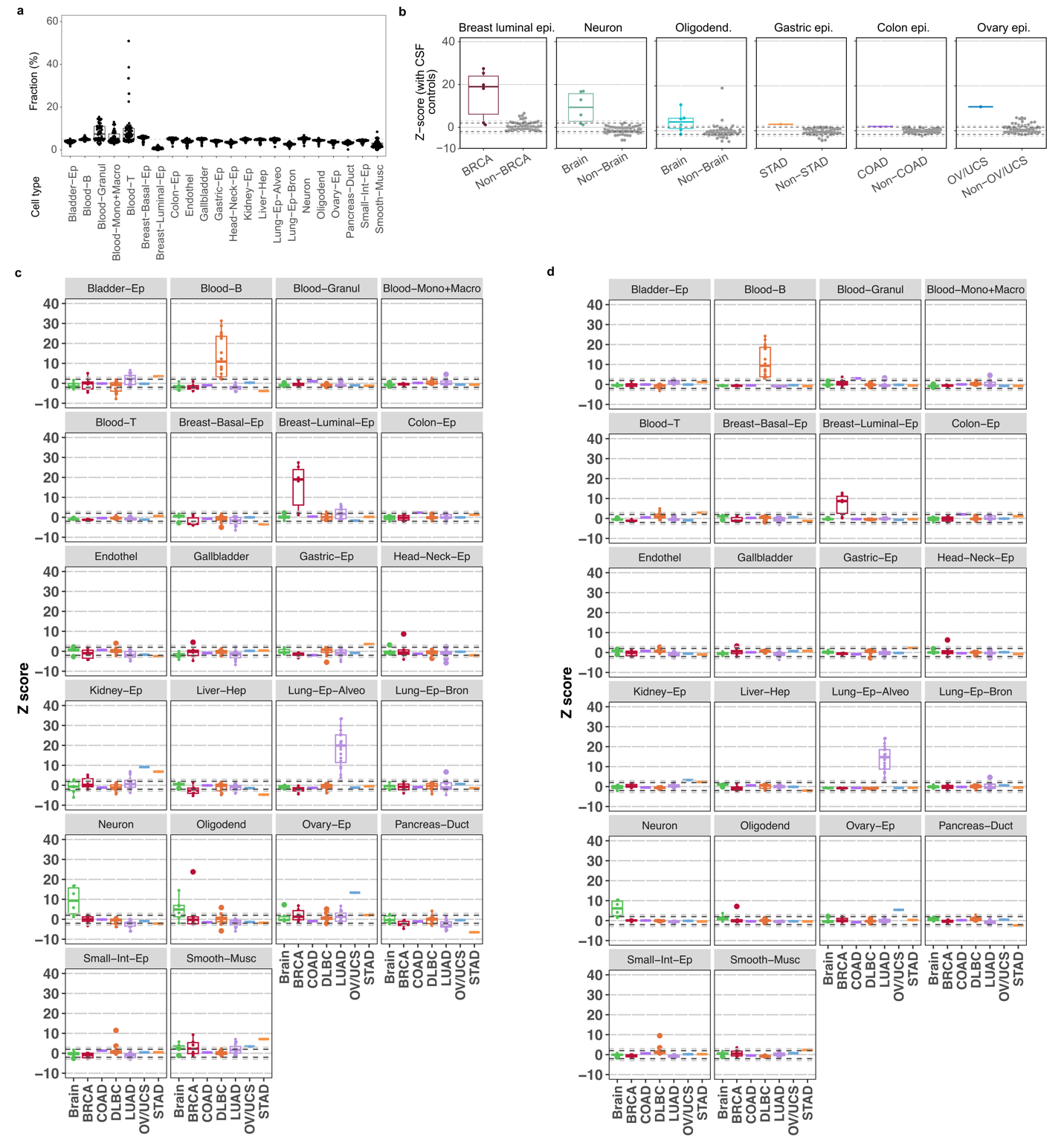


**Supplementary Figure S6:** Deconvolution of CSF samples. **a**, Deconvoluted cell type proportions in CSF negative controls (n = 50). Four extreme outliers with high T-cell percentages were excluded in the following analyses. **b,** Z-scores of the cell-of-origin in less common CNS metastases and primary CNS tumors (labeled Brain) in 56 CSF tumor samples. **c**, Z-scores of all 22 deconvoluted cell types across 56 CSF tumor samples as normalized by the negative controls. **d,** Z-scores of all 22 cell types, but normalized against non-target tumors. Brain, primary brain tumors; STAD, stomach adenocarcinoma; OV, ovarian cancer; UCS, uterine carcinosarcoma.


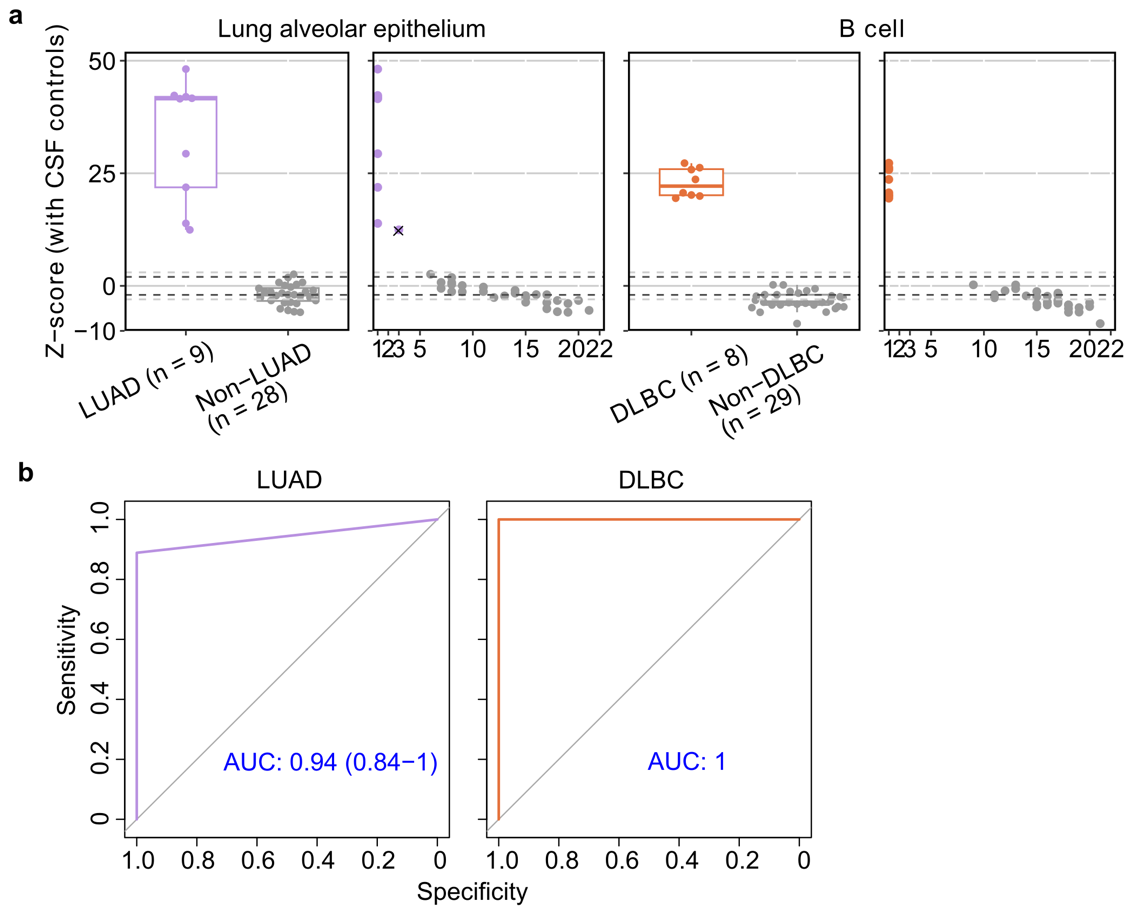


**Supplementary Figure S7:** Deconvolution of FFPE tissue DNA samples**. a**, Z-scores of the cell-of-origin of representative FFPE tissue DNA samples: lung alveolar epithelium for LUADs (vs. non-LUADs) and B cell for DLBCs (vs. non-DLBCs). The purple dots and box indicate LUAD, and the orange dots and box indicate DLBC. The x-axis in the boxplots indicates the target and non-target tumors, and the x-axis in the scatter plots indicates the z-score rankings of the target and non-target tumors. The black and gray dashed lines are at z-scores of 2 and 3, respectively. **b**, ROC curves for deconvolution classification for LUAD and DLBC. Negative controls and two indeterminates were excluded. The ‘x’ indicates one misclassified case.

**Supplementary Tables**

**See the spreadsheets.**

**Supplementary Protocol**

This protocol was adapted in part using the EM-seq kit from NEB. Please see manufacturer’s instructions for any updates.

**1.1. DNA Preparation**

**1.2. End Prep of Fragmented DNA**

1.2.1. On ice, mix the following components in a sterile nuclease-free PCR tube: Fragmented DNA 25 µl, (green) NEBNext Ultra II End Prep Reaction Buffer 3.5 µl, (green) NEBNext Ultra II End Prep Enzyme Mix 1.5 µl. Total volume 30 µl.

1.2.2. Mix thoroughly by vortexing 1–2 seconds or by pipetting up and down at least 10 times and centrifuge briefly. Note: It is important to mix well. The presence of a small amount of bubbles will not interfere with the performance.

1.2.3. Place in a thermal cycler with the heated lid set to ≥ 75°C or on, and run the following program: 30 minutes at 20 °C, 30 minutes at 65 °C, and hold at 4 °C.

**1.3. Ligation of Adaptor A**

1.3.1. On ice, add the following components directly to the End Prep reaction mixture and mix well: End Prep reaction mixture 30 µl, Adaptor A 1.0 µl, (red) NEBNext Ligation Enhancer 0.5 µl, (red) NEBNext Ultra II Ligation Master Mix 15 µl. Total volume 46.5 µl.

1.3.2. Mix thoroughly by vortexing 1-2 seconds or by pipetting up and down at least 10 times and centrifuge briefly. Caution: The Ligation Master Mix is viscous. Care should be taken to ensure adequate mixing of the ligation reaction, as incomplete mixing will result in reduced ligation efficiency. The presence of a small amount of bubbles will not interfere with performance.

1.3.3. Place in a thermal cycler, and run the following program with the heated lid off: 15 minutes at 20 °C, and hold at 4 °C.

**1.4. Clean-Up of Adaptor-A-Ligated DNA**

1.4.1. Vortex the Sample Purification Beads to resuspend.

1.4.2. Add 50 µl of resuspended Purification Beads to each sample. Mix well by pipetting up and down at least 10 times. Be careful to expel all of the liquid out of the tip during the last mix.

1.4.3. Incubate samples on bench top for at least 5 minutes at room temperature.

1.4.4. Place the tubes against an appropriate magnetic stand to separate the beads from the supernatant.

1.4.5. After 5 minutes (or when the solution is clear), carefully remove and discard the supernatant. Be careful not to disturb the beads that contain DNA targets.

1.4.6. Add at least 90 μl of freshly prepared 80% ethanol to the tubes while on the magnetic stand. Incubate at room temperature for 30 seconds, and then carefully remove and discard the supernatant. Be careful not to disturb the beads that contain DNA targets.

1.4.7. Repeat the ethanol wash once for a total of two washes.

1.4.8 Remove all visible liquid after the second wash using a p10 pipette tip.

1.4.9. Air dry the beads for up to 2 minutes while the tubes are on the magnetic stand with the lid open. Caution: Do not over-dry the beads.

1.4.10. Remove the tubes from the magnetic stand. Elute the DNA target from the beads by adding 17 µl of Elution Buffer.

1.4.11. Mix well by pipetting up and down 10 times. Incubate for at least 1 minute at room temperature. If necessary, quickly spin the sample to collect the liquid from the sides of the tube before placing back on the magnetic stand.

1.4.12. Place the tube on the magnetic stand. After 3 minutes (or whenever the solution is clear), transfer the supernatant to a new PCR tube.

**1.5. End Repair after Adapter A Purification**

1.5.1. Remove residual phosphates using 1 µL Shrimp Alkaline Phosphatase (rSAP, 1,000 units/ml, M0371L, NEB) in 2 µL of 10X NEB buffer 2.

1.5.2. Incubate at 37 °C for 1h and 65 °C for 10 min.

1.5.3. Add 10 U of MspI (R0106T, NEB), and incubate for 30 min at room temperature.

1.5.4. Add 1 µL of Klenow master mix for end repair: incubate at 30 °C for 20 min, 37 °C for 20 min, and 65 °C for 20 min. The master mix consistes of 1.3 µL of Klenow (M0212M, NEB), 2 µL of 10X NEB buffer 2, 5 µL of NTPs mix (composed of 40 µL dATP, 4 µL dCTP, and 4 µL dGTP, all at 10 mM), and 11.7 µL of water.

**1.6. Ligation of Adaptor B**

1.6.1. On ice, add the following components directly to the End Prep reaction mixture and mix well: End Prep reaction mixture 21 µl, Adaptor B 1 µl, (red) NEBNext Ligation Enhancer 0.4 µl, (red) NEBNext Ultra II Ligation Master Mix 12 µl.

1.6.2. Mix thoroughly by vortexing 1-2 seconds or by pipetting up and down at least 10 times and centrifuge briefly.

1.6.3. Place in a thermal cycler, and run the following program with the heated lid off: 15 minutes at 20 °C, and hold at 4 °C.

**1.7. Clean-Up of Adaptor-B-Ligated DNA**

1.7.1. Vortex the Purification Beads to resuspend.

1.7.2. Add 36 µl of resuspended Purification Beads to each sample. Mix well by pipetting up and down at least 10 times. Be careful to expel all of the liquid out of the tip during the last mix.

1.7.3. Incubate samples on bench top for at least 5 minutes at room temperature.

1.7.4. Place the tubes against an appropriate magnetic stand to separate the beads from the supernatant.

1.7.5. After 5 minutes (or when the solution is clear), carefully remove and discard the supernatant. Be careful not to disturb the beads that contain DNA targets.

1.7.6. Add at least 90 μl of freshly prepared 80% ethanol to the tubes while on the magnetic stand. Incubate at room temperature for 30 seconds, and then carefully remove and discard the supernatant. Be careful not to disturb the beads that contain DNA targets.

1.7.7. Repeat the ethanol wash once for a total of two washes.

1.7.8 Remove all visible liquid after the second wash using a p10 pipette tip.

1.7.9. Air dry the beads for up to 2 minutes while the tubes are on the magnetic stand with the lid open. Caution: Do not over-dry the beads.

1.7.10. Remove the tubes from the magnetic stand. Elute the DNA target from the beads by adding 28 µl of Elution Buffer.

1.7.11. Mix well by pipetting up and down 10 times. Incubate for at least 1 minute at room temperature. If necessary, quickly spin the sample to collect the liquid from the sides of the tube before placing back on the magnetic stand.

1.7.12. Place the tube on the magnetic stand. After 3 minutes (or whenever the solution is clear), transfer the supernatant to a new PCR tube.

**1.8. Protection of 5-Methylcytosines and 5-Hydroxymethylcytosines**

1.8.1. Prepare TET2 Buffer. Add 400 µl of (yellow) TET2 Reaction Buffer to one tube of (yellow) TET2 Reaction Buffer Supplement and mix well by vortexing and/ or pipette mixing until the TET2 Reaction Buffer Supplement is completely in solution. Spin down before use. Write date on tube. Critical: The reconstituted buffer should be stored at -20 °C and discarded after 4 months.

1.8.2. On ice, add the following components directly to the adaptor ligated DNA: adaptor ligated DNA 28 µl, (yellow) TET2 Reaction Buffer (TET2 Reaction Buffer Supplement reconstituted in TET2 Reaction Buffer) 10 µl, (yellow) Oxidation Supplement 1 µl, (yellow) DTT 1 µl, (yellow) Oxidation Enhancer 1 µl, (yellow) TET2 4 µl. Total volume 45 µl. Mix thoroughly by vortexing for 1–2 seconds or by pipetting up and down at least 10 times and centrifuge briefly. 5mC/5hmC oxidation is initiated by the addition of the Fe(II) Solution to the reaction in the next step.

1.8.3. Dilute the 500 mM (yellow) Fe(II) Solution by adding 1 µl to 1,249 µl of water. Mix well by vortexing. On ice, combine diluted (yellow) Fe(II) Solution and DNA from Section 1.8.2. as described below: Reaction Mixture 45 µl, Diluted (yellow) Fe(II) Solution (Step 1.8.3.) 5 µl. Total volume 50 µl. Mix thoroughly by vortexing for 1-2 seconds or by pipetting up and down at least 10 times and centrifuge briefly.

1.8.4. Place in a thermal cycler and run the following program with the heated lid set to ≥ 45 °C or on: 1 hour at 37 °C, and hold at 4 °C.

1.8.5. Transfer the samples to ice and add 1 µl of (yellow) Stop Reagent: Protected DNA (Step 1.8.4.) 50 µl and (yellow) Stop Reagent 1 µl. Total volume 51 µl. Mix thoroughly by vortexing for 1-2 seconds or by pipetting up and down at least 10 times and centrifuge briefly.

1.8.6. Place in a thermal cycler with the heated lid set to ≥ 45 °C or on and run the following program: 30 minutes at 37 °C, and hold at 4 °C.

**1.9. Clean-Up of TET2 Converted DNA**

1.9.1. Vortex Purification Beads to resuspend.

1.9.2. Add 84 µl of resuspended Purification Beads to each sample. Mix well by pipetting up and down at least 10 times. Be careful to expel all of the liquid out of the tip during the last mix.

1.9.3. Incubate samples on bench top for at least 5 minutes at room temperature.

1.9.4. Place the tubes against an appropriate magnetic stand to separate the beads from the supernatant.

1.9.5. After 5 minutes (or when the solution is clear), carefully remove and discard the supernatant. Be careful not to disturb the beads that contain DNA targets (Caution: do not discard the beads).

1.9.6. Add at least 90 μl of freshly prepared 80% ethanol to the tubes while on the magnetic stand. Incubate at room temperature for 30 seconds, and then carefully remove and discard the supernatant. Be careful not to disturb the beads that contain DNA targets.

1.9.7. Repeat the wash once for a total of two washes.

1.9.8. Remove all visible liquid after the second wash using a p10 pipette tip.

1.9.9. Air dry the beads for up to 2 minutes while the tubes are on the magnetic stand with the lid open.

1.9.10. Remove the tubes from the magnetic stand. Elute the DNA target from the beads by adding 17 µl of Elution Buffer.

1.9.11. Mix well by pipetting up and down 10 times. Incubate for at least 1 minute at room temperature. If necessary, quickly spin the sample to collect the liquid from the sides of the tube before placing back on the magnetic stand.

1.9.12. Place the tube on the magnetic stand. After 3 minutes (or whenever the solution is clear), transfer 12 µl of the supernatant to a new PCR tube.

**1.10. Denaturation of DNA using Formamide**

1.10.1. Pre-heat thermal cycler to 85 °C with the heated lid set to ≥ 105 °C or on.

1.10.2. Add 3 µl Formamide to the 12 µl of protected DNA (Step 1.9.12). Mix thoroughly by vortexing for 1-2 seconds or by pipetting up and down at least 10 times, centrifuge briefly.

1.10.3. Incubate at 85 °C for 10 minutes in the pre-heated thermal cycler.

1.10.4. Critical Step: Immediately place in cooling block on ice and allow the sample to fully cool (~ 2 minutes) before proceeding to Section 1.11.

**1.11. Deamination of Cytosines**

1.11.1. On ice, add the following components to the denatured DNA: Denatured DNA 15 µl, Nuclease-free water 51 µl, (orange) APOBEC Reaction Buffer 7.5 µl, (orange) BSA 0.75 µl, (orange) APOBEC 0.75 µl.

1.11.2. Mix thoroughly by vortexing for 1-2 seconds or by pipetting up and down at least 10 times and centrifuge briefly.

1.11.3. Place in a thermal cycler and run the following program with the heated lid set to ≥ 45 °C or on: 3 hours at 37 °C, and hold at 4 °C.

**1.12. Clean-Up of Deaminated DNA**

1.12.1. Vortex Sample Purification Beads to resuspend.

1.12.2. Add 90 µl of resuspended Purification Beads to each sample. Mix well by pipetting up and down at least 10 times. Be careful to expel all of the liquid out of the tip during the last mix.

1.12.3. Incubate samples on bench top for at least 5 minutes at room temperature.

1.12.4. Place the tubes against an appropriate magnetic stand to separate the beads from the supernatant.

1.12.5. After 5 minutes (or when the solution is clear), carefully remove and discard the supernatant. Be careful not to disturb the beads that contain DNA targets (Caution: do not discard the beads).

1.12.6. Add at least 90 μl of freshly prepared 80% ethanol to the tubes while on the magnetic stand. Incubate at room temperature for 30 seconds, and then carefully remove and discard the supernatant. Be careful not to disturb the beads that contain DNA targets.

1.12.7. Repeat the wash once for a total of two washes.

1.12.8. Remove all visible liquid after the second wash using a p10 pipette tip.

1.12.9. Air dry the beads for up to 60 seconds while the tubes are on the magnetic stand with the lid open. Do not overdry beads at this critical step.

1.12.10. Remove the tubes from the magnetic stand. Elute the DNA target from the beads by adding 15 µl of Elution Buffer.

1.12.11. Mix well by pipetting up and down 10 times. Incubate for at least 1 minute at room temperature. If necessary, quickly spin the sample to collect the liquid from the sides of the tube before placing back on the magnetic stand.

1.12.12. Place the tube on the magnetic stand. After 3 minutes (or whenever the solution is clear), transfer 15 µl of the supernatant to a new PCR tube.

**1.13. PCR Amplification**

1.13.1. On ice, add the following components to the deaminated DNA from Step 1.12.12.: Deaminated DNA 7.5 µl, EM-seq Index Primer or similar 2.5 µl, NEBNext Q5U Master Mix 10 µl with spike-in of SYBR Gold (7.5 uL of 1:100 diluted SYBR into 1.25 mL Master Mix). Repeat this into a second well.

1.13.2. Mix thoroughly by vortexing or by pipetting up and down at least 10 times, centrifuge briefly.

1.13.3. Place the tube in a thermal cycler with the heated lid set to 105 °C and perform PCR amplification using the following cycling conditions: Initial Denaturation at 98 °C for 30 seconds; Denaturation at 98 °C for 10 seconds, Annealing at 62 °C for 30 seconds, Extension at 65 °C for 60 seconds, and total 20 cycles. Final Extension at 65 °C for 5 minutes. Hold at 25 °C. Stopping Point: Samples can be stored overnight at either 4 °C in the thermal cycler or at -20 °C in the freezer.

**1.14. Clean-Up of Amplified Libraries**

**1.15. Library Quantification**

Run on a Qubit dsDNA HS assay for quantification. Dilute to the desired concentration for sequencing.

**References**

1. Gu,W., Talevich,E., Hsu,E., Qi,Z., Urisman,A., Federman,S., Gopez,A., Arevalo,S., Gottschall,M., Liao,L., *et al.* (2021) Detection of cryptogenic malignancies from metagenomic whole genome sequencing of body fluids. *Genome Medicine*, **13**, 98.

2. Gu,W., Rauschecker,A.M., Hsu,E., Zorn,K.C., Sucu,Y., Federman,S., Gopez,A., Arevalo,S., Sample,H.A., Talevich,E., *et al.* (2021) Detection of Neoplasms by Metagenomic Next-Generation Sequencing of Cerebrospinal Fluid. *JAMA Neurol*, **78**, 1355–1366.

3. Gu,W., Deng,X., Lee,M., Sucu,Y.D., Arevalo,S., Stryke,D., Federman,S., Gopez,A., Reyes,K., Zorn,K., *et al.* (2021) Rapid pathogen detection by metagenomic next-generation sequencing of infected body fluids. *Nat Med*, **27**, 115–124.

4. Gao,Y., Zhao,H., An,K., Liu,Z., Hai,L., Li,R., Zhou,Y., Zhao,W., Jia,Y., Wu,N., *et al.* (2022) Whole‐genome bisulfite sequencing analysis of circulating tumour DNA for the detection and molecular classification of cancer. *Clinical & Translational Med*, **12**, e1014.

5. Maros,M.E., Capper,D., Jones,D.T.W., Hovestadt,V., von Deimling,A., Pfister,S.M., Benner,A., Zucknick,M. and Sill,M. (2020) Machine learning workflows to estimate class probabilities for precision cancer diagnostics on DNA methylation microarray data. *Nat Protoc*, **15**, 479–512.

6. Koelsche,C., Schrimpf,D., Stichel,D., Sill,M., Sahm,F., Reuss,D.E., Blattner,M., Worst,B., Heilig,C.E., Beck,K., *et al.* (2021) Sarcoma classification by DNA methylation profiling. *Nat Commun*, **12**, 498.

7. Capper,D., Jones,D.T.W., Sill,M., Hovestadt,V., Schrimpf,D., Sturm,D., Koelsche,C., Sahm,F., Chavez,L., Reuss,D.E., *et al.* (2018) DNA methylation-based classification of central nervous system tumours. *Nature*, **555**, 469–474.

8. Talevich,E., Shain,A.H., Botton,T. and Bastian,B.C. (2016) CNVkit: Genome-Wide Copy Number Detection and Visualization from Targeted DNA Sequencing. *PLOS Computational Biology*, **12**, e1004873.

9. Caggiano,C., Celona,B., Garton,F., Mefford,J., Black,B.L., Henderson,R., Lomen-Hoerth,C., Dahl,A. and Zaitlen,N. (2021) Comprehensive cell type decomposition of circulating cell-free DNA with CelFiE. *Nat Commun*, **12**, 2717.

10. Loyfer,N., Magenheim,J., Peretz,A., Cann,G., Bredno,J., Klochendler,A., Fox-Fisher,I., Shabi-Porat,S., Hecht,M., Pelet,T., *et al.* (2023) A DNA methylation atlas of normal human cell types. *Nature*, **613**, 355–364.

11. Loyfer,N., Rosenski,J. and Kaplan,T. (2024) wgbstools: A computational suite for DNA methylation sequencing data representation, visualization, and analysis. 10.1101/2024.05.08.593132.

12. Shimizu,H., Horii,A., Sunamura,M., Motoi,F., Egawa,S., Unno,M. and Fukushige,S. (2011) Identification of epigenetically silenced genes in human pancreatic cancer by a novel method “microarray coupled with methyl-CpG targeted transcriptional activation” (MeTA-array). *Biochemical and Biophysical Research Communications*, **411**, 162–167.

13. Qiao,H., Yin,H., Feng,Y. and Tang,H. (2022) Pan-cancer analysis reveals the relationship between RCSD1 immune infiltration and clinical prognosis in human tumors. *Front. Immunol.*, **13**, 1008778.

14. Yao,X., Deng,Y., Zhou,J., Jiang,L. and Song,Y. (2023) Expression Pattern and Prognostic Analysis of Branched-Chain Amino Acid Catabolism-Related Genes in Non-Small Cell Lung Cancer. *Front. Biosci. (Landmark Ed)*, **28**, 107.

15. Hwang,S.-H., Kim,K.U., Kim,J.-E., Kim,H.-H., Lee,M.K., Lee,C.H., Lee,S.-Y., Oh,T. and An,S. (2011) Detection of *HOXA9* gene methylation in tumor tissues and induced sputum samples from primary lung cancer patients. *Clinical Chemistry and Laboratory Medicine*, **49**, 699–704.
